# The effect of general practice contact on cancer stage at diagnosis in Aboriginal and non-Aboriginal residents of New South Wales

**DOI:** 10.1007/s10552-023-01727-6

**Published:** 2023-06-17

**Authors:** David Banham, David Roder, Sandra Thompson, Anna Williamson, Freddie Bray, David Currow

**Affiliations:** 1 Cancer Statistics and Information Division, Cancer Institute of New South Wales, St Leonards, NSW Australia; 2 Cancer Epidemiology and Population Health, University of South Australia, Adelaide, Australia; 3 WA Centre for Rural Health, University of Western Australia, Perth, Australia; 4 The Sax Institute, Sydney, Australia; 5 Cancer Surveillance Branch, International Agency for Research On Cancer, Lyon, France; 6 Faculty of Science, Medicine and Health, University of Wollongong, Wollongong, Australia

**Keywords:** Indigenous status, Cancer disparities, Comorbidity, Socioeconomic position, Primary care, Local stage at diagnosis

## Abstract

**Purpose:**

Older age, risks from pre-existing health conditions and socio-economic disadvantage are negatively related to the prospects of an early-stage cancer diagnosis. With older Aboriginal Australians having an elevated prevalence of these underlying factors, this study examines the potential for the mitigating effects of more frequent contact with general practitioners (GPs) in ensuring local-stage at diagnosis.

**Methods:**

We compared the odds of local vs. more advanced stage at diagnosis of solid tumours according to GP contact, using linked registry and administrative data. Results were compared between Aboriginal (*n* = 4,084) and non-Aboriginal (*n* = 249,037) people aged 50 + years in New South Wales with a first diagnosis of cancer in 2003–2016.

**Results:**

Younger age, male sex, having less area-based socio-economic disadvantage, and fewer comorbid conditions in the 12 months before diagnosis (0–2 vs. 3 +), were associated with local-stage in fully-adjusted structural models. The odds of local-stage with more frequent GP contact (14 + contacts per annum) also differed by Aboriginal status, with a higher adjusted odds ratio (aOR) of local-stage for frequent GP contact among Aboriginal people (aOR = 1.29; 95% CI 1.11–1.49) but not among non-Aboriginal people (aOR = 0.97; 95% CI 0.95–0.99).

**Conclusion:**

Older Aboriginal Australians diagnosed with cancer experience more comorbid conditions and more socioeconomic disadvantage than other Australians, which are negatively related to diagnosis at a local-cancer stage. More frequent GP contact may act to partly offset this among the Aboriginal population of NSW.

**Supplementary Information:**

The online version contains supplementary material available at 10.1007/s10552-023-01727-6.

## Background

Cancer is a significant and increasing public health problem among older Aboriginal Australians, adding to existing health disparities [1]. A preponderance of more lethal cancer types and poorer survival for Aboriginal people following cancer diagnosis [2–5] is seen in New South Wales (NSW) [6], the Northern Territory [7], Queensland [8] and South Australia [9, 10].

Cancer stage at diagnosis is a key prognostic indicator of cancer survival, with local-stage, being a favourable indicator, yet Aboriginal people are less likely than other Australians to be diagnosed with local as opposed to more advanced cancers [2, 9, 11–13].

While comorbidity is associated with later stage at cancer diagnosis [14], as with other First Nations people [15, 16], Aboriginal Australians experience an elevated prevalence of comorbid conditions [17], starting at an early age [8, 12, 18–21]. Older age, which is associated with increased comorbidity in the population [22], and in cancer patients specifically [16], is also linked to later stage diagnoses [23] [24]. Socio-economic disadvantage is associated with cancer onset at a younger age and more comorbidity, an observation seen in Australia and elsewhere [25, 26]. For example, the prevalence of major chronic conditions as part of multi-comorbidities has been reported in Australia to range from around 14% in geographic areas of least disadvantage to 24% in areas of most disadvantage [22].

With all people in Australia having access to the universal health care system, it would be expected, if adjusting effectively for all other factors influencing general practice (GP) attendance, that people who were older and with more comorbidity would access GP services more frequently [27]. Aboriginal Australians regard GPs as their usual source of health care, and on average, their need for primary health care is greater than that of other Australians [28]. However, the Royal Australian College of General Practitioners reports that Aboriginal contact with GPs is comparatively low [29].

We hypothesize that supporting increased GP contact by Aboriginal people might increase medical surveillance, earlier detection, and tailoring of cancer care to better meet individual and community needs [30]. To date, few population studies have investigated pathways to diagnosis of cancer according to GP or other primary health care contact in a disease-specific context [26, 31].

There is a need to improve our understanding of the characteristics of Aboriginal people diagnosed with cancer, including their experience with comorbidity, use of GP services, and any influence of GP use on stage of cancer at diagnosis [32]. We have compared the experiences of older Aboriginal Australians with those of other older Australians in adjusted as well as unadjusted analyses.

## Aims

The principal objectives are to:

1. undertake a population-based retrospective cohort study to investigate the pathway from comorbidity to local-stage at cancer diagnosis in older Aboriginal residents of New South Wales first diagnosed in 2003–2016;
2. compare the Aboriginal pathway to local-stage diagnosis with the corresponding pathway for non-Aboriginal contemporaries;
3. investigate the potential for frequent GP contact to mitigate any negative effects of Aboriginal status on likelihood of local-stage of cancer at diagnosis; and
4. facilitate communication of study results to Aboriginal and non-Aboriginal people via the use of simple, illustrative paths within a Structural Equation Modelling approach.

## Methods

### Study setting and data sources

The setting was New South Wales (NSW), Australia’s most populous state, with a population of over 7.5 million in 2016. Self-identified Aboriginal people (including Torres Strait Islanders in this study) comprised 3.4% of the NSW population with one in six Aboriginal people (*n* = 37,293) being aged 50 + years [33]. Australians have a universal health care system that provides comprehensive primary care, mostly through general practitioners (GPs) and hospital-based services.

Data sources for the de-identified linked dataset used in this study included the NSW Cancer Registry (NSWCR) [34], NSW Admitted Patient Data Collection (APDC) [35], and Medicare Benefits Schedule (MBS). This study was part of a research program entitled “*The cancer and healthy ageing in Aboriginal NSW older Generations (CHANGES)”.* The program was funded by the National Health and Medical Research Council (NH&MRC) as a collaborative initiative aimed at informing evidence-based, integrated cancer care pathways for older Aboriginal Australians.

We retrospectively constructed a cohort of NSW residents aged 50 years or more at time of first cancer diagnosis during the study period from July 2003 to December 2016, using NSWCR data. The NSWCR is a continuous statutory data collection of all mandatorily reported invasive cancers diagnosed in NSW residents. The NSWCR records diagnosis date, primary site, and summary degree of spread of solid cancers, along with demographic information including age and sex, for all cancer diagnoses.

Two area-level indicators were available for analysis, including: (1) socio-economic disadvantage based on the Australian Bureau of Statistics Index of Relative Socio-economic Disadvantage (IRSD) [36] where we used Quintile 5 to represent the most disadvantaged areas and Quintiles 1 the least disadvantaged; and (2) geographic residential remoteness based on the Accessibility/Remoteness Index of Australia (ARIA +) [37]. ARIA areas were classified as Major cities, Inner regional, Outer regional, and Remote and Very Remote areas.

Registry records were person-linked with discharge record extracts from the APDC from July 2001, which included ICD-10AM diagnostic codes for all NSW hospitals. This enabled counts of comorbid conditions of relevance to the Elixhauser comorbidity index [38]. To examine GP contact, we used counts of MBS claims from the national collection.

NSWCR and APDC records were probabilistically linked by the Centre for Health Record Linkage using a privacy-protecting protocol. A study-specific “Project Person Number” was used to join individuals’ records without disclosing personal identifiers. Cumulative numbers of false positive and false negative linkages were measured at less than 5 per 1000. The Australian Institute of Health and Welfare subsequently linked NSWCR and APDC hospital records with MBS records. The linked data were lodged and analysed within the Secure Unified Research Environment SURE [39], a purpose built research infrastructure facility.

### Study cohort

The Study cohort comprised NSW residents aged 50 + years at the time of first cancer diagnosis in the study period. We restricted the cohort to those with a first occurring cancer with a primary site of lung, breast (female), cervix, pancreas, liver, colon, rectum, prostate, and head and neck due to their importance in the Aboriginal population. Using this approach, we reported tumour characteristics, a methodology consistent with that used by the NSW Ministry of Health and Cancer Institute in its flagship annual reporting of *Reporting for Better Cancer Outcomes* [40]. We grouped cohort members by Aboriginal status, using an earlier reported ‘weight of evidence’ algorithm [41] specifically designed for use with NSWCR data.

**Fig. 1:**
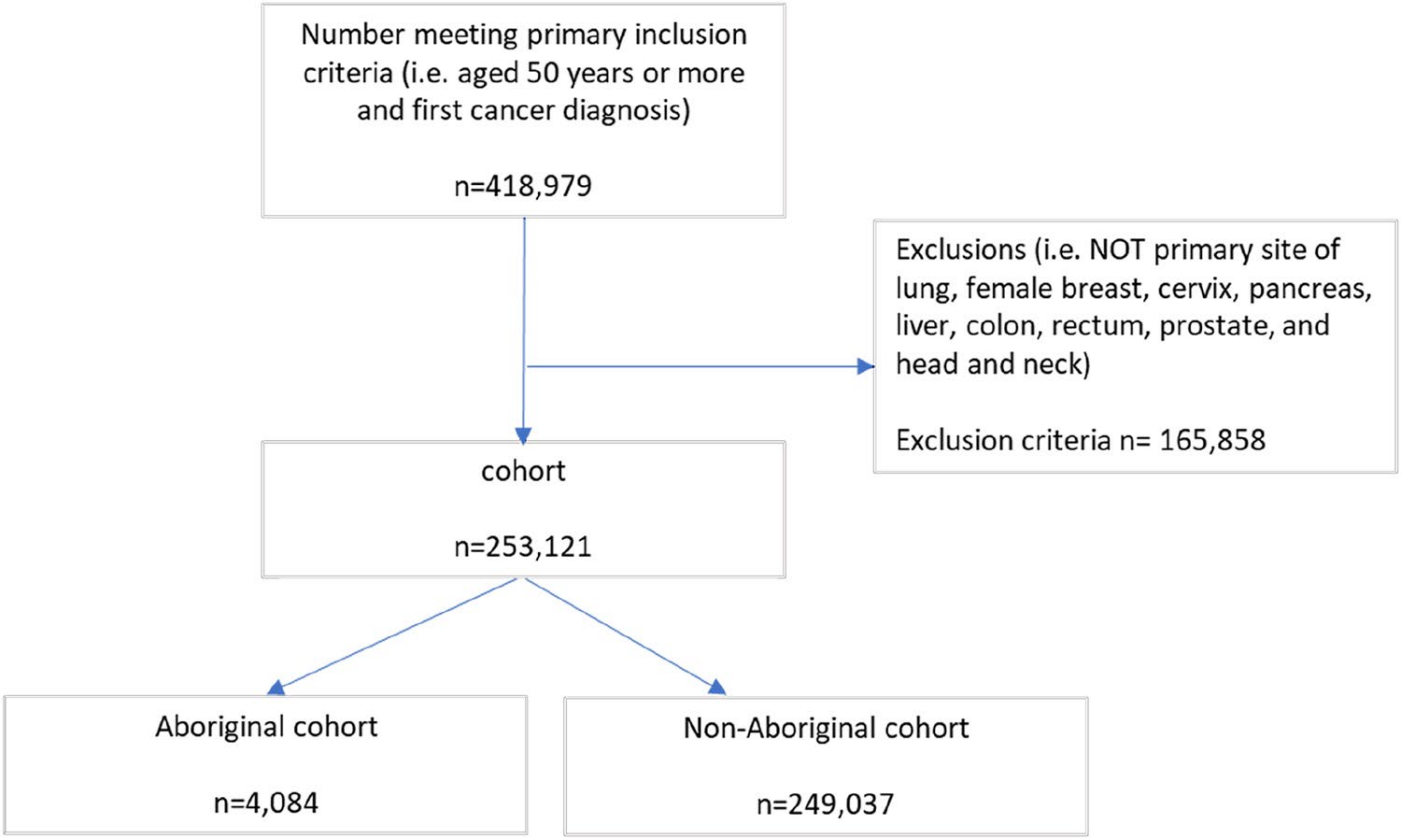
Cohort construction

The cohort construction is as shown in Fig. 1.

### Outcomes

We studied two main variables. The first was the number of hospital-recorded comorbid health conditions as a predictor variable, using conditions of relevance to the Elixhauser comorbidity index [38]. We excluded cancer-related conditions from the index to ensure that the comorbidity conditions examined were distinct from the cancer. A 12-month look-back period from diagnosis was used, consistent with earlier Australian research [42, 43] and to optimise use of the available linked cancer and hospital records. Summed numbers of conditions ranged from 0 (no hospital coded comorbidity) to a maximum of 26. Using quintile distributions as a guide we dichotomised comorbidities as 0 to 2 conditions (= 0) and three or more (= 1) to give a reasonable number of cases for comparison above and below the cut-off.

Number of GP contacts was our second main variable (the main predictor), comprising numbers of MBS records for GP consultation occurring in the 12-months leading to cancer diagnosis. We defined GP consultations as records involving a professional attendance and a description referring to “GP” or “General Practitioner”, or reference to an Aboriginal and Torres Strait Islander health assessment (Item 715). Using the interquartile distribution of these GP counts as a guide, we dichotomised the data as 0–13 GP contacts (= 0) or 14 contacts or more (= 1). Again, this cut-off gave a reasonable number for comparison above and below the cut-off.

Local stage of cancer at diagnosis was our third main variable (the key outcome). The NSWCR recorded complete summary staging information for the study period. We dichotomised stage into local cancer (= 1) and more advanced spread (regional, distant or unknown = 0).

### Study variables

These included NSWCR variables for primary cancer site and stage, and for: patient age at diagnosis classified as 50–69 years (= 0) or 70 + years (= 1); sex (male = 0, female = 1); residential area socioeconomic disadvantage (most disadvantaged quintile (= 1), lesser disadvantage (= 0); residential remoteness (Major city and Inner regional (= 0), Outer regional and Remote (= 1), and residential proximity to borders with other jurisdictions (i.e., Local Health Districts of Northern NSW, Southern NSW, Murrumbidgee, Albury and Far West (= 1) and all others (= 0)). Many border residents were known to have some hospitalisation outside NSW, thereby recording artificially reduced comorbidity in the NSW data. This variable allowed for adjustment for border residence and sensitivity analysis according to whether border residents were included in the analysis.

### Statistical methods

Analyses were undertaken using Stata 16.0 [44] within the SURE environment [39]. Descriptive cross-tabulations described Aboriginal status by: age at diagnosis (years); sex; residential area of socioeconomic disadvantage; geographic remoteness; living adjacent to the border of another state or territory; comorbidity status; number of GP contacts; stage (degree of spread) at diagnosis; and primary cancer site.

Aboriginal and non-Aboriginal people were also compared for each of these variables using logistic regression, deriving unadjusted and adjusted odds ratios (OR) with their 95% confidence intervals (95%CIs). This approach was repeated within Aboriginal and non-Aboriginal people separately to describe bivariate distributions of socio-demographic and cancer variables along the structural pathway. Separate tables were used for comorbid conditions, GP contacts, and local-stage. All potential covariates related to the main variables at the univariate level were simultaneously evaluated for inclusion in our multivariable structural models. Variables were removed in a stepwise manner where they did not contribute to statistically significant associations with each outcome during testing of the structural pathway.

We then specified our structural model which included directional relationships based on the empirical evidence reviewed and also including GP contacts as a potential mitigating factor for likelihood of non-local stage [45]. Our approach included testing associations for comorbid condition numbers, numbers of GP contacts, and stages at diagnosis for each cohort using a series of multivariable logit models.

**Fig. 2:**
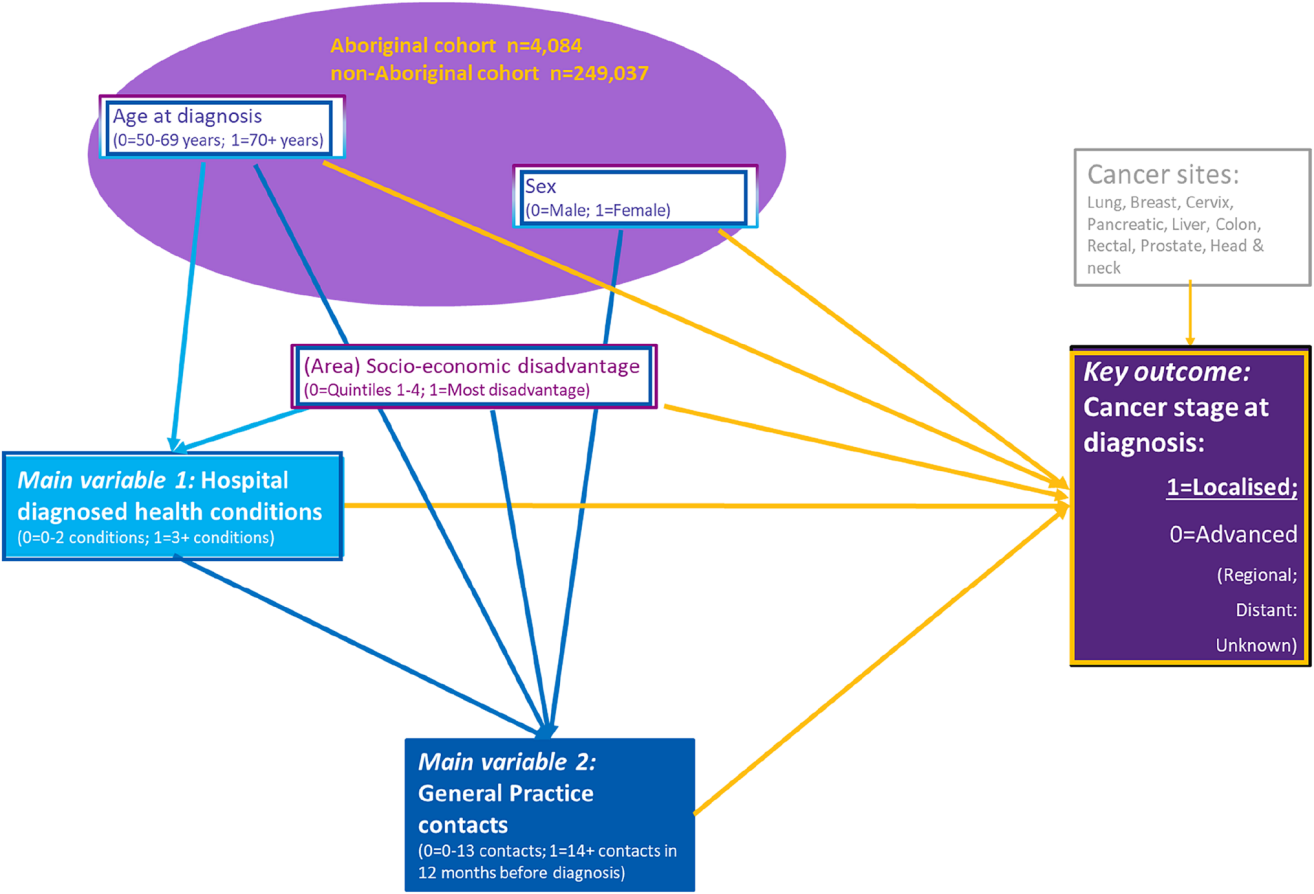
Conceptual model of the structural equation pathway from comorbid conditions and general practice contact to cancer stage at diagnosis

Figure 2 presents a visual representation of this directional structural model, culminating in diagnosis of local-stage cancer [46]. We tabled the fully adjusted model in the text along with the Hosmer–Lemeshow statistics [47] to indicate goodness-of-fit. To support methodological transparency, and improve communication of results [48], visualisation of the modelled estimates was provided to supplement table results.

In supplementing the analysis, we tested the sensitivity of our structural model in three ways. First, we included and excluded border area residents, then undertook stratified analyses, using full models, stratified by each primary cancer site (i.e.: lung, female breast, cervix, pancreas, liver, colon, rectum, prostate, and head and neck). In particular, models were examined for signs of GP contacts affecting earlier cancer detection. Finally, we recognised the potential information loss by dichotomising variables [49] within our parsimonious model and reran the models using age, comorbid conditions numbers and GP consults as continuous measures and discrete area disadvantage quintiles.

## Results

Among the 253,121 people with cancer aged 50 years or over and diagnosed in 2003–2016 in this study, 4,084 (1.6%) were Aboriginal (Fig. 1). Table 1 summarises the descriptive characteristics of Aboriginal and non-Aboriginal participants. The Aboriginal cohort had a significantly younger profile with 68.0% vs. 52.4% being aged under 70 years. Females were marginally more common in Aboriginal (43.1%) than non-Aboriginal (40.1%) members.

**Table 1:** Socio-demographic, service use and cancer characteristics of Aboriginal and non-Aboriginal study cohorts

	Aboriginal cohort		Non-Aboriginal cohort		Odds Ratio (unadjusted)	Lower 95%CI	Upper 95%CI	*p*
	*n*	Column %	*n*	Column %				
Total	4,084	100.0	249,037	100.0				
*Age group at diagnosis*								
50 to 69 years	2,777	68.0	130,527	52.4	1.00	Reference		
70 or more years	1,307	32.0	118,510	47.6	0.52	0.49	0.55	< 0.001
*Sex*								
Male	2,322	56.9	149,158	59.9	1.00	Reference		
Female	1,762	43.1	99,879	40.1	1.13	1.06	1.21	< 0.001
*Area level Index of Relative Socio-economic Disadvantage (IRSD)*								
Least disadvantage Quintiles 1 to 4	1,191	29.2	135,355	54.4	1.00	Reference		
Most disadvantage Quintile 5	2,893	70.8	113,682	45.6	2.89	2.70	3.10	< 0.001
*Geographic remoteness*								
Major cities & Inner regional	3,113	76.2	227,811	91.5	1.00	Reference		
Outer regional & remote	971	23.8	21,226	8.5	3.35	3.11	3.60	< 0.001
*Reside near state border*								
No	3,331	81.6	214,705	86.2	1.00	Reference		
Yes	753	18.4	34,332	13.8	1.41	1.31	1.53	< 0.001
*Comorbid conditions (Elixhauser)*								
0 to 2 conditions	3,360	82.3	221,450	88.9	1.00	Reference		
3 or more conditions	724	17.7	27,587	11.1	1.73	1.59	1.88	< 0.001
*General Practice (GP) contact*								
0 to 13 consults	2,795	68.4	186,590	74.9	1.00	Reference		
14 or more consults	1,289	31.6	62,447	25.1	1.38	1.29	1.47	< 0.001
*Stage at diagnosis*								
Advanced*	2,650	68.4	149,434	60.0	1.00	Reference		
Local	1,434	31.6	99,603	40.0	0.81	0.76	0.87	< 0.001
*Primary site (Reporting for Better Cancer Outcomes)*								
Lung	960	23.5	39,391	15.8	1.91	1.71	2.14	< 0.001
Breast (female)	708	17.3	46,993	18.9	1.18	1.05	1.33	0.006
Cervix	53	1.3	1,521	0.6	2.73	2.05	3.65	< 0.001
Pancreatic	184	4.5	10,708	4.3	1.35	1.13	1.60	0.001
Liver	192	4.7	6,290	2.5	2.39	2.02	2.84	< 0.001
Colon	454	11.1	35,605	14.3	1.00	Reference		
Rectal	258	6.3	18,761	7.5	1.08	0.92	1.26	0.336
Prostate	1,020	25.0	80,242	32.2	1.00	0.89	1.11	0.956
Head and neck	255	6.2	9,526	3.8	2.10	1.80	2.45	< 0.001

*Includes regional, distant and unknown/unstageable

The OR for Aboriginal vs. non-Aboriginal patients: living in the most socio-economic disadvantaged than less disadvantaged areas was 2.89 (95%CI 2.70, 3.10); and living in more remote than inner regional and major city areas was 3.35 (95%CI 3.11, 3.60). Despite their younger age, Aboriginal patients had higher levels of comorbid conditions with 17.7% vs. 11.1% for non-Aboriginal people having 3 + conditions recorded during their hospital stays. In the 12-months leading to diagnosis, Aboriginal people used GP services more frequently (31.6% vs. 25.1% had 14 + GP contacts). Local-stage was comparatively less likely among Aboriginal patients at OR = 0.81 (95%CI 0.76, 0.87). Primary site of cancer also varied by Aboriginal status with lung cancer being the most frequent within the Aboriginal cohort (23.5%) vs. prostate cancer (32.2%) among non-Aboriginal members.

Table 2 shows the bivariate distribution of socio-demographic and cancer variables by comorbid condition categories (the first of our structural pathway outcomes) for Aboriginal and non-Aboriginal cohorts respectively. Increased age was associated with increased comorbidity (3 + conditions), with OR = 3.09 (95%CI 3.01, 3.18) for ages 70 + vs. 50–69 years. The increase with age occurred particularly in the non-Aboriginal cohort, with the OR lower in the Aboriginal cohort because relatively more younger patients (< 70 years) also had 3 or more conditions (14.8% in the Aboriginal and 9.4% in the non-Aboriginal cohort). Higher comorbidity numbers were observed among non-Aboriginal males. Area disadvantage was associated with increased comorbidity among Aboriginal and non-Aboriginal patients with OR = 1.49 (95%CI 1.23, 1.80) and OR = 1.41 (95%CI 1.37–1.45) respectively living in most disadvantaged vs. less disadvantaged areas. The likelihood of more frequent GP contacts increased among patients with 3 + comorbidities. Notably, Aboriginal patients without elevated comorbid numbers also had more frequent visits (28.5%) compared to their non-Aboriginal contemporaries (22.7%).

**Table 2:** Unadjusted, bivariate relationships between comorbid condition numbers and the characteristics of Aboriginal and non-Aboriginal cohorts

	Aboriginal cohort								Non-Aboriginal cohort							
	0 to 2 conditions	Column %	3 or more conditions	Column %	Odds Ratio (unadjusted)	Lower 95%CI	Upper 95%CI	p	0 to 2 conditions	Column %	3 or more conditions	Column %	Odds Ratio (unadjusted)	Lower 95%CI	Upper 95%CI	p
Total	3,360	100.0	724	100.0					221,450	100.0	27,587	100.0				
Age group at diagnosis																
50 to 69 years	2,364	70.4	413	57.0	1.00	Reference			122,628	55.4	7,899	28.6	1.00	Reference		
70 or more years	996	29.6	311	43.0	1.79	1.52	2.11	< 0.001	98,822	44.6	19,688	71.4	3.09	3.01	3.18	< 0.001
Sex																
Male	1,904	56.7	418	57.7	1.00	Reference			132,293	59.7	16,865	61.1	1.00	Reference		
Female	1,456	43.3	306	42.3	0.96	0.81	1.13	0.599	89,157	40.3	10,722	38.9	0.94	0.92	0.97	< 0.001
Area level Index of Relative Socio-economic Disadvantage (IRSD)																
Least disadvantage Quintiles 1 to 4	1,026	30.5	165	22.8	1.00	Reference			122,459	55.3	12,896	46.7	1.00	Reference		
Most disadvantage Quintile 5	2,334	69.5	559	77.2	1.49	1.23	1.80	< 0.001	98,991	44.7	14,691	53.3	1.41	1.37	1.45	< 0.001
Geographic remoteness																
Major cities & Inner regional	2,568	76.4	545	75.3	1.00	Reference			202,269	91.3	25,542	92.6	1.00	Reference		
Outer regional & remote	792	23.6	179	24.7	1.06	0.88	1.28	0.509	19,181	8.7	2,045	7.4	0.84	0.81	0.89	< 0.001
Reside near state border																
No	2,721	81.0	610	84.3	1.00	Reference			190,200	85.9	24,505	88.8	1.00	Reference		
Yes	639	19.0	114	15.7	0.80	0.64	0.99	0.040	31,250	14.1	3,082	11.2	0.77	0.74	0.80	< 0.001
General Practice (GP) contact																
0 to 13 consults	2,401	71.5	394	54.4	1.00	Reference			171,159	77.3	15,431	55.9	1.00	Reference		
14 or more consults	959	28.5	330	45.6	2.10	1.78	2.47	< 0.001	50,291	22.7	12,156	44.1	2.68	2.61	2.75	< 0.001
Stage at diagnosis																
Advanced*	2,142	71.5	508	70.2	1.00	Reference			129,721	77.3	19,713	71.5	1.00	Reference		
Local	1,218	28.5	216	29.8	0.75	0.63	0.89	0.001	91,729	22.7	7,874	28.5	0.56	0.55	0.58	< 0.001
Primary cancer site																
Lung	729	21.7	231	31.9	0.87	0.68	1.13	0.293	31,953	14.4	7,438	27.0	0.96	0.92	0.99	0.021
Breast (female)	654	19.5	54	7.5	0.23	0.16	0.32	< 0.001	45,330	20.5	1,663	6.0	0.15	0.14	0.16	< 0.001
Cervix	41	1.2	12	1.7	0.81	0.41	1.58	0.531	1,353	0.6	168	0.6	0.51	0.43	0.60	< 0.001
Pancreatic	124	3.7	60	8.3	1.33	0.92	1.93	0.131	8,306	3.8	2,402	8.7	1.19	1.13	1.25	< 0.001
Liver	102	3.0	90	12.4	2.43	1.71	3.45	< 0.001	4,170	1.9	2,120	7.7	2.09	1.97	2.22	< 0.001
Colon	333	9.9	121	16.7	1.00	Reference			28,646	12.9	6,959	25.2	1.00	Reference		
Rectal	203	6.0	55	7.6	0.75	0.52	1.07	0.113	16,604	7.5	2,157	7.8	0.53	0.51	0.56	< 0.001
Prostate	953	28.4	67	9.3	0.19	0.14	0.27	< 0.001	76,615	34.6	3,627	13.1	0.19	0.19	0.20	< 0.001
Head & neck	221	6.6	34	4.7	0.42	0.28	0.64	< 0.001	8,473	3.8	1,053	3.8	0.51	0.48	0.55	< 0.001
*Includes regional, distant and unknown/unstageable																

Table 3 shows numbers of GP contacts, the intermediary process measure, and the distribution of socio-demographic and cancer variables. In both Aboriginal and non-Aboriginal cohorts, the OR for 14 + GP visits was higher among older patients aged 70 + years than younger patients, and particularly so for non-Aboriginal than Aboriginal patients, i.e.: ORs = 2.21 (95%CI 2.17, 2.25) and 1.69 (95%CI 1.47, 1.94) respectively. In both cohorts, females had a higher likelihood of frequent 14 + GP contacts at ORs = 1.15 (95%CI 1.01, 1.32) and 1.13 (95%CI 1.11,1.15) respectively.

**Table 3:** Unadjusted, bivariate relationships between general practice (GP) contacts and the characteristics of Aboriginal and non-Aboriginal cohorts

	Aboriginal cohort								Non-Aboriginal cohort							
	0 to 13 GP contacts	Column %	14 or more GP contacts	Column %	Odds Ratio (unadjusted)	Lower 95%CI	Upper 95%CI	p	0 to 13 GP contacts	Column %	14 or more GP contacts	Column %	Odds Ratio (unadjusted)	Lower 95%CI	Upper 95%CI	p
Total	2,795	100.0	1,289	100.0					186,590	100.0	62,447	100.0				
Age group at diagnosis																
50 to 69 years	2,004	71.7	773	60.0	1.00	Reference			106,914	57.3	23,613	37.8	1.00	Reference		
70 or more years	791	28.3	516	40.0	1.69	1.47	1.94	< 0.001	79,676	42.7	38,834	62.2	2.21	2.17	2.25	< 0.001
Sex																
Male	1,620	58.0	702	54.5	1.00	Reference			113,160	60.6	35,998	57.6	1.00	Reference		
Female	1,175	42.0	587	45.5	1.15	1.01	1.32	0.036	73,430	39.4	26,449	42.4	1.13	1.11	1.15	< 0.001
Area level Index of Relative Socio-economic Disadvantage (IRSD)																
Least disadvantage Quintiles 1 to 4	876	31.3	315	24.4	1.00	Reference			105,240	56.4	30,115	48.2	1.00	Reference		
Most disadvantage Quintile 5	1,919	68.7	974	75.6	1.41	1.21	1.64	< 0.001	81,350	43.6	32,332	51.8	1.39	1.36	1.41	< 0.001
Geographic remoteness																
Major cities & Inner regional	2,131	76.2	982	76.2	1.00	Reference			169,747	91.0	58,064	93.0	1.00	Reference		
Outer regional & remote	664	23.8	307	23.8	1.00	0.86	1.17	0.966	16,843	9.0	4,383	7.0	0.76	0.73	0.79	< 0.001
Reside near state border																
No	2,270	81.2	1,061	82.3	1.00	Reference			159,135	85.3	55,570	89.0	1.00	Reference		
Yes	525	18.8	228	17.7	0.93	0.78	1.10	0.402	27,455	14.7	6,877	11.0	0.72	0.70	0.74	< 0.001
Comorbid conditions (Elixhauser)																
0 to 2 conditions	2,401	85.9	959	74.4	1.00	Reference			171,159	91.7	50,291	80.5	1.00	Reference		
3 or more conditions	394	14.1	330	25.6	2.10	1.78	2.47	< 0.001	15,431	8.3	12,156	19.5	2.68	2.61	2.75	< 0.001
Stage at diagnosis																
Advanced*	1,838	65.8	812	63.0	1.00	Reference			109,824	58.9	39,610	63.4	1.00	Reference		
Local	957	34.2	477	37.0	1.13	0.98	1.29	0.085	76,766	41.1	22,837	36.6	0.82	0.81	0.84	< 0.001
Primary site (Reporting for Better Cancer Outcomes)																
Lung	603	21.6	357	27.7	1.27	1.01	1.62	0.045	25,292	13.6	14,099	22.6	1.50	1.45	1.54	< 0.001
Breast (female)	517	18.5	191	14.8	0.80	0.61	1.03	0.082	37,299	20.0	9,694	15.5	0.70	0.67	0.72	< 0.001
Cervix	41	1.5	12	0.9	0.63	0.32	1.23	0.179	1,196	0.6	325	0.5	0.73	0.64	0.83	< 0.001
Pancreatic	104	3.7	80	6.2	1.66	1.16	2.36	0.005	6,661	3.6	4,047	6.5	1.63	1.56	1.71	< 0.001
Liver	110	3.9	82	6.4	1.60	1.13	2.27	0.008	3,942	2.1	2,348	3.8	1.60	1.51	1.69	< 0.001
Colon	310	11.1	144	11.2	1.00	Reference			25,935	13.9	9,670	15.5	1.00	Reference		
Rectal	182	6.5	76	5.9	0.90	0.64	1.25	0.530	14,837	8.0	3,924	6.3	0.71	0.68	0.74	< 0.001
Prostate	742	26.5	278	21.6	0.81	0.63	1.03	0.080	64,265	34.4	15,977	25.6	0.67	0.65	0.69	< 0.001
Head & neck	186	6.7	69	5.4	0.80	0.57	1.12	0.194	7,163	3.8	2,363	3.8	0.88	0.84	0.93	< 0.001
*Includes regional, distant and unknown/unstageable																

Irrespective of Aboriginal status, patients residing in areas of most disadvantage had more frequent GP contacts than those in less disadvantaged areas. Residents of border areas had less frequent GP contact. Aboriginal patients diagnosed with local cancer tended, more than those with more advanced cancer, to have 14 + GP contacts per annum. While there was no a priori power calculation, this approached, but did not achieve, statistical significance (OR = 1.13 (95%CI 0.98, 1.29); p = 0.085). In contrast, non-Aboriginal patients with local-stage diagnosis were less likely to have 14 + GP contacts (OR = 0.82 (95%CI 0.81, 0.84)).

Associations with the principal outcome of local cancer are shown in Table 4. Younger age was associated with increased odds of diagnosis at a local-stage, ORs of 0.85 (95%CI 0.74, 0.98) and 0.65 (95%CI 0.64, 0.67) among Aboriginal and non-Aboriginal people respectively. In both cohorts, those living in areas of most (compared with less) disadvantage had a similar lower likelihood of localised cancers at diagnosis, with ORs of 0.80 (95%CI 0.69, 0.92) and 0.78 (95%CI 0.77, 0.80) for Aboriginal and non-Aboriginal respectively.

**Table 4:** Unadjusted, bivariate relationships between stage at diagnosis and the Aboriginal and non-Aboriginal cohorts by cohort characteristics

	Aboriginal cohort								Non-Aboriginal cohort							
	Advanced* stage	Column %	Local stage	Column %	Odds Ratio (unadjusted)	Lower 95%CI	Upper 95%CI	*p*	Advanced* stage	Column %	Local stage	Column %	Odds Ratio (unadjusted)	Lower 95% CI	Upper 95% CI	*p*
Total	2650	100.0	1434	100.0					149,434	100.0	99,603	100.0				
Age group at diagnosis																
50 to 69 years	1769	66.8	1008	70.3	1.00	Reference			72,049	48.2	58,478	58.7	1.00	Reference		
70 or more years	881	33.2	426	29.7	0.85	0.74	0.98	0.021	77,385	51.8	41,125	41.3	0.65	0.64	0.67	< 0.001
Sex																
Male	1457	55.0	865	60.3	1.00	Reference			89,118	59.6	60,040	60.3	1.00	Reference		
Female	1193	45.0	569	39.7	0.80	0.71	0.92	0.001	60,316	40.4	39,563	39.7	0.97	0.96	0.99	0.001
Area level Index of Relative Socio-economic Disadvantage (IRSD)																
Least disadvantage Quintiles 1 to 4	729	27.5	462	32.2	1.00	Reference			77,595	51.9	57,760	58.0	1.00	Reference		
Most disadvantage Quintile 5	1921	72.5	972	67.8	0.80	0.69	0.92	0.002	71,839	48.1	41,843	42.0	0.78	0.77	0.80	< 0.001
Geographic remoteness																
Major cities & Inner regional	2013	76.0	1100	76.7	1.00	Reference			136,536	91.4	91,275	91.6	1.00	Reference		
Outer regional & remote	637	24.0	334	23.3	0.96	0.82	1.12	0.593	12,898	8.6	8,328	8.4	0.97	0.94	0.99	0.018
Reside near state border																
No	2139	80.7	1192	83.1	1.00	Reference			128,295	85.9	86,410	86.8	1.00	Reference		
Yes	511	19.3	242	16.9	0.85	0.72	1.01	0.058	21,139	14.1	13,193	13.2	0.93	0.91	0.95	< 0.001
Comorbid conditions (Elixhauser)																
0 to 2 conditions	1838	69.4	957	66.7	1.00	Reference			109,824	73.5	76,766	77.1	1.00	Reference		
3 or more conditions	812	30.6	477	33.3	0.75	0.63	0.89	0.001	39,610	26.5	22,837	22.9	0.56	0.55	0.58	< 0.001
General Practice (GP) contact																
0 to 13 consults	1838	69.4	957	66.7	1.00	Reference			109,824	73.5	76,766	77.1	1.00	Reference		
14 or more consults	812	30.6	477	33.3	1.13	0.98	1.29	0.085	39,610	26.5	22,837	22.9	0.82	0.81	0.84	< 0.001
Primary site (Reporting for Better Cancer Outcomes)																
Lung	785	29.6	175	12.2	0.62	0.48	0.81	< 0.001	31,974	21.4	7417	7.4	0.54	0.52	0.56	< 0.001
Breast (female)	366	13.8	342	23.8	2.60	2.01	3.36	< 0.001	21,838	14.6	25,155	25.3	2.69	2.61	2.77	< 0.001
Cervix	40	1.5	13	0.9	0.90	0.47	1.75	0.766	1,011	0.7	510	0.5	1.18	1.06	1.31	0.003
Pancreatic	159	6.0	25	1.7	0.44	0.27	0.70	0.001	9,030	6.0	1678	1.7	0.43	0.41	0.46	< 0.001
Liver	115	4.3	77	5.4	1.86	1.31	2.66	0.001	3,568	2.4	2722	2.7	1.78	1.69	1.88	< 0.001
Colon	334	12.6	120	8.4	1.00	Reference			24,934	16.7	10,671	10.7	1.00	Reference		
Rectal	188	7.1	70	4.9	1.04	0.73	1.46	0.839	12,244	8.2	6517	6.5	1.24	1.20	1.29	< 0.001
Prostate	480	18.1	540	37.7	3.13	2.46	3.99	< 0.001	38,706	25.9	41,536	41.7	2.51	2.44	2.57	< 0.001
Head and neck	183	6.9	72	5.0	1.10	0.78	1.54	0.604	6,129	4.1	3397	3.4	1.30	1.23	1.36	< 0.001

*Includes regional, distant and unknown/unstageable

From these bivariate associations, we inferred the direction and components of the structural model (Fig. 2). The first stage included age and area socioeconomic status as predictors of hospital coded comorbidity. Predictors of our second and intermediate process measure (frequency of GP contact) were age, sex, socio-economic disadvantage and comorbidity. Ultimately, the predictors examined for local-stage were age, sex, socioeconomic disadvantage, comorbidity and GP contacts.

Age and disadvantage were associated with the pathway to comorbidity (Table 5). Older age group was associated with 3 + compared with fewer comorbid conditions and more so among non-Aboriginal at adjusted OR = 3.05 (95%CI 2.96, 3.13) compared with adjusted OR = 1.81 (95%CI 1.53, 2.14) for Aboriginal members. Living in areas of greatest rather than lesser disadvantage was also related to 3 + comorbid conditions in Aboriginal members at adjusted OR = 1.52 (95%CI 1.26, 1.84) and in non-Aboriginal members at adjusted OR = 1.33 (95%CI 1.30, 1.37).

**Table 5:** Multivariable models of influences on main pathway variables of comorbidity and GP contacts, and key outcome of local-stage cancer diagnosis among Aboriginal and non-Aboriginal cohorts

	Aboriginal cohort				Non-Aboriginal cohort			
	Odds Ratio (adjusted)	Lower 95%CI	Upper 95%CI	p	Odds Ratio (adjusted)	Lower 95%CI	Upper 95%CI	p
Main variable 1: 3 or more comorbid conditions								
Age group at diagnosis								
50 to 69 years	1.00	Reference			1.00	Reference		
70 or more years	1.81	1.53	2.14	< 0.001	3.05	2.96	3.13	< 0.001
Area level Index of Relative Socio-economic Disadvantage (IRSD)								
Least disadvantage Quintiles 1 to 4	1.00	Reference			1.00	Reference		
Most disadvantage Quintile 5	1.52	1.26	1.84	< 0.001	1.33	1.30	1.37	< 0.001
Main variable 2: 14 or more GP consults								
Age group at diagnosis								
50 to 69 years	1.00	Reference			1.00	Reference		
70 or more years	2.02	1.75	2.33	< 0.001	2.39	2.35	2.44	< 0.001
Sex								
Male	1.00	Reference			1.00	Reference		
Female	1.15	1.00	1.32	0.056	1.09	1.07	1.12	< 0.001
Area level Index of Relative Socio-economic Disadvantage (IRSD)								
Least disadvantage Quintiles 1 to 4	1.00	Reference			1.00	Reference		
Most disadvantage Quintile 5	1.11	0.95	1.30	0.181	1.10	1.08	1.12	< 0.001
Comorbid conditions (Elixhauser)								
0 to 2 conditions	1.00	Reference			1.00	Reference		
3 or more conditions	2.45	2.07	2.90	< 0.001	2.72	2.64	2.79	< 0.001
Key outcome: Local-stage at diagnosis								
Age group at diagnosis								
50 to 69 years	1.00	Reference			1.00	Reference		
70 or more years	0.82	0.72	0.95	0.008	0.70	0.68	0.71	< 0.001
Sex								
Male	1.00	Reference			1.00	Reference		
Female	0.80	0.70	0.91	< 0.001	0.97	0.96	0.99	< 0.001
Area level Index of Relative Socio-economic Disadvantage (IRSD)								
Least disadvantage Quintiles 1 to 4	1.00	Reference			1.00	Reference		
Most disadvantage Quintile 5	0.81	0.70	0.93	< 0.001	0.81	0.80	0.82	< 0.001
Comorbid conditions (Elixhauser)								
0 to 2 conditions	1.00	Reference			1.00	Reference		
3 or more conditions	0.73	0.61	0.88	0.001	0.64	0.62	0.65	< 0.001
GP consults								
0 to 13 consults	1.00	Reference			1.00	Reference		
14 or more consults	1.29	1.11	1.49	0.001	0.97	0.95	0.99	0.006

Higher frequency of GP contacts or 14 + per year was observed in the older age group, females, residents of most disadvantaged areas and those with higher comorbidity counts. These differences were apparent in both Aboriginal and non-Aboriginal cohorts and of similar magnitude, apart from by age where the OR in Aboriginal patients appeared to be somewhat lower at adjusted OR = 2.02 (95%CI 1.75, 2.33) compared with adjusted OR = 2.39 (95%CI 2.35, 2.44)) for their non-Aboriginal counterparts.

Age, sex, area disadvantage, comorbid condition numbers and GP contact frequency all were associated with the pathway to cancer diagnosis at a local-stage. Increased age, female sex, increased disadvantage and comorbidities all were associated with a lower likelihood of cancer diagnosis at a local-stage. The adjusted effect sizes indicated by these differences were similar within the Aboriginal and non-Aboriginal cohorts.

The association of frequent GP contacts (14 + GP contacts per year) with local-stage differed markedly between the Aboriginal and non-Aboriginal cohorts. Among non-Aboriginal cancer patients, frequent contacts were associated with a marginally lower odds of local cancer at adjusted OR = 0.97 (95%CI 0.95, 0.99), after taking account of the aforementioned factors. An opposite association with more frequent GP contacts was observed among Aboriginal patients where increased GP contacts were associated with a higher likelihood of local cancer at adjusted OR = 1.29 (95%CI 1.11, 1.49). A diagrammatic form of the enumerated pathway is provided in Supplementary Figure S1.

Hosmer–Lemeshow goodness of fit was satisfactory for our modelled evaluation of comorbid condition numbers, general practice contacts and local-stage diagnosis within the Aboriginal cohort with χ^2^(2) = 0.06, p = 0.969; χ^2^(2) = 1.0; χ^2^(6) = 4.57, p = 0.600 respectively. Testing the sensitivity of modelled results to removal from the analysis of patients living in border areas gave very similar results for associations of frequent GP contacts with local cancer at diagnosis, the adjusted ORs being 1.26 (95%CI 1.08, 1.48) and 0.95 (95%CI 0.93, 0.97) among Aboriginal and non-Aboriginal patients respectively. Details are provided in Supplementary Table S1.

**Fig. 3:**
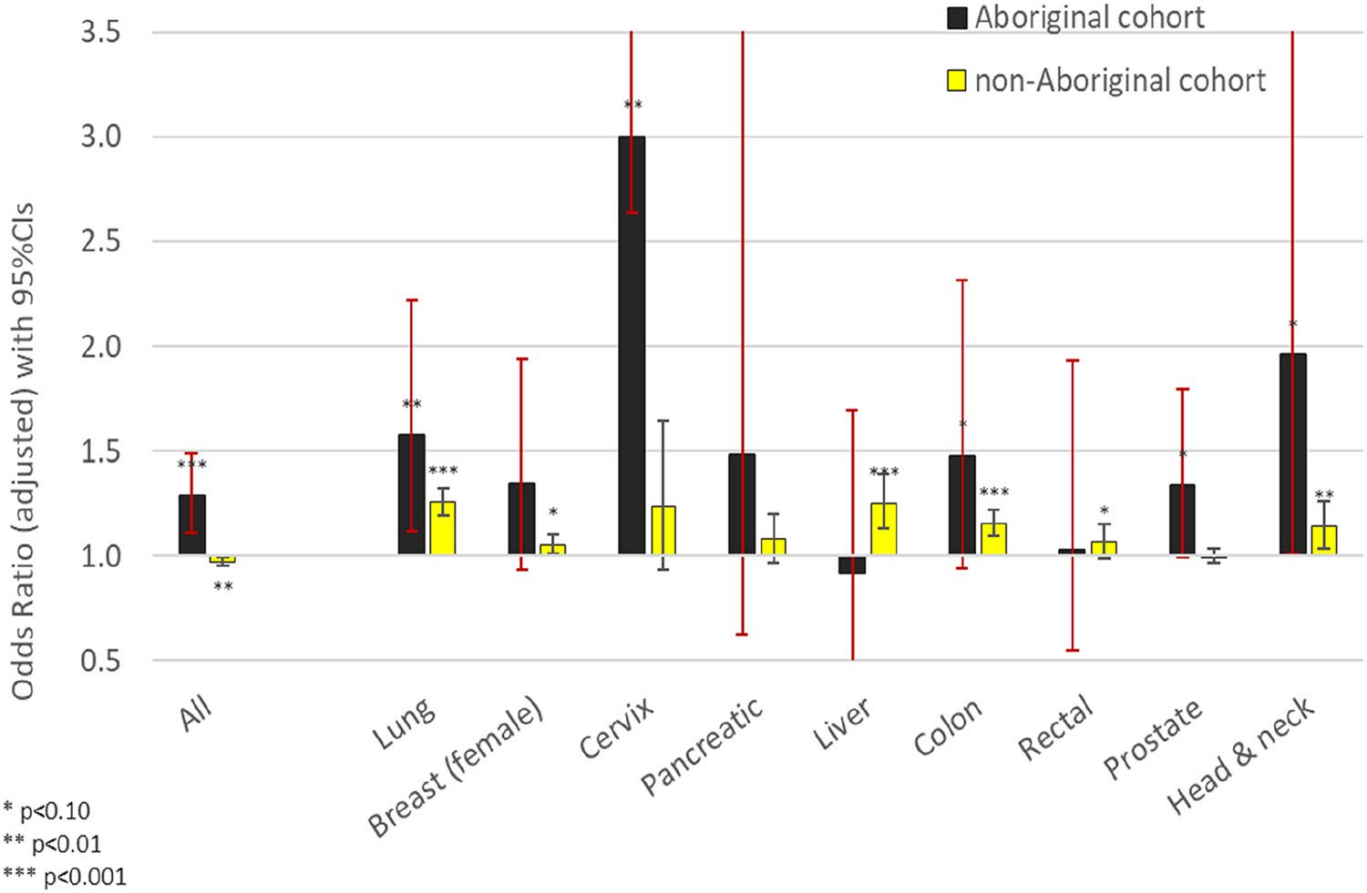
The influence of 14 or more GP contacts in the 12-months before local-stage cancer diagnosis by cancer site and Aboriginal status

We further stratified our structural equation modelling for the 9 major cancer sites. Results by cancer site and Aboriginal status are provided in Supplementary Table S2. The adjusted odds ratios for the association of frequent GP contacts with diagnosis at a local-stage are summarised in Fig. 3. Frequent GP contact was positively associated with local-stage in both cohorts for cancers of the lung, colon, and head and neck. Positive associations were also observed among Aboriginal patients for cervical and prostate cancers; and, among non-Aboriginal patients for breast, liver and rectal cancers.

Finally, we tested the sensitivity of our modelling of local-stage diagnosis and replaced dichotomised variables with continuous measures of age, comorbid condition numbers and GP contact, and all socioeconomic disadvantage quintiles in Supplementary Table S3. The results support the concurrent influence of those variables on local-stage diagnosis among the cohort of Aboriginal people whereby increasing age and comorbidity numbers lowered the odds of local stage diagnosis while more GP consults and less disadvantage increased the odds of local stage diagnosis.

## Discussion

### Study aims

Assessment of the initial step in the pathway to comorbidity indicated the important influences of age and socio-economic disadvantage on comorbidity risk. Disadvantage was more pronounced among Aboriginal than non-Aboriginal patients, as was comorbidity, particularly at a younger age. Comorbidity was negatively associated with detection of cancer at a local-stage. By comparison, high GP contact was positively associated with local-stage in Aboriginal patients. The reasons are not clear, but a possible explanation is that high GP contact in Aboriginal patients reduces delay in cancer diagnosis, with commensurate impacts on risk of cancer death. Issues of trust of health care professionals and accessibility of health services are further issues which have been identified as important with respect to delayed diagnosis of cancer in Aboriginal people [50].

The question arises whether promoting high GP contact would be beneficial in terms of primary prevention and preventing cancers developing to advanced stages before diagnosis. Our results indicate comorbidity, along with age and socio-economic disadvantage, has a negative effect on detecting cancer at a localised stage, and that high GP contact partially offsets that effect. The mediating effect of high GP contact could arise in several ways the first of which is by encouraging participation in cancer screening. Increased screening participation would be consistent with the characteristics of Aboriginal specific primary health care services focussed not only on treatment and management, but on prevention, health promotion and addressing social health determinants [51]. Also, more culturally relevant relationships between patient and practitioner may lead to more consistent care leading to increased surveillance and earlier identification of cancer symptoms or changes of concern. More research is needed to confirm this finding and understand the underlying mechanisms, for example by examining the degree of use *and type* of primary health care service attended. If that research is productive, further questions will consider how best to promote GP contact and in what context.

Our statistical methodology was Structural Equation Modelling, which we found enabled presentation of results in a structured form that could be expressed diagrammatically and readily understood by both Aboriginal and non-Aboriginal members of the community. The results prompted discussion and encouraged consideration of further research needs and service development.

### Comorbidity

Our findings are consistent with broader cohort analyses, including those undertaken in the CanDAD project [21]. While one in three Australians aged 45 to 64 years report having at least two chronic health conditions (23), the figures at about 50% in people aged 65 years or more were higher in the present study in the 12 months leading to cancer diagnosis [22].

### Frequent GP contact

Our results, based on MBS records for the 12 months leading to cancer diagnosis, indicate that 25% had 14 + GP contacts. This figure can be compared with ABS national survey data for 2020 indicating that 15% of Australians aged 55 + years or more visited a GP 12 + times in the previous 12 months [52]. We found a higher proportion visited a GP 14 + times in 12 months in Quintile 5 (most disadvantaged) at 34% among Aboriginal and 28% among non-Aboriginal people in Quintile 5, as compared with 26% and 22% respectively in Q1-4. Meanwhile a RACGP report indicated more GP contacts per year in most and least disadvantaged people respectively (30).

### Local-stage

We found 35% of Aboriginal vs. 40% of non-Aboriginal older patients had local-stage at diagnosis. These proportions are lower than whole of population averages, consistent with a decrease in local-stage with older age. A NSW study in 2001–2007 covering all ages and a broader range of primary sites, found 40% had local-stage among Aboriginal and 47% among non-Aboriginal patients (52). Corresponding proportions with local-stage were: 37% for Aboriginal vs. 50% for non-Aboriginal patients in a SA matched cohort study across ages [9]; 50% in Queensland overall and with a lower percentage in Aboriginal patients [8]; 38% and 45% in Aboriginal and non-Aboriginal patients respectively in an updated Queensland cohort [12]; and in the Northern Territory, 34% vs. 44% for Aboriginal and non-Aboriginal cases respectively for lung, breast, cervix, and bowel cancers collectively [53].

We also found that 34% had local-stage among Aboriginal patients living in outer regional and remote areas of NSW vs. 39% in urban inner regional areas. Within the Aboriginal people in NSW, the percentage with local-stage was 36% for rural/remote vs. 43% for urban dwellers [11]. While rurality is important, these data showed area level socio-economic disadvantage exhibited an even larger influence on the diagnosis of local-stage cancers among Aboriginal the study cohort.

### Supplementary analyses

These data, presented in the Supplementary Tables, suggested: (1) similar results irrespective of whether border LHDs were included in the modelling; and (2) generally consistent positive associations of GP contact with earlier stage in Aboriginal patients for most primary cancer sites.

The latter, stratified analyses showed the relative frequency of cancer site influenced the overall, positive effect of frequent GP contact. For example, lung cancer diagnosis was relatively more common within the Aboriginal cohort where it made up almost one-quarter of cases. Frequent GP contact significantly improved the odds of diagnosing lung cancer at local stage after accounting for the damaging effects of age, comorbid conditions and disadvantage. A similar effect was observed among the relatively smaller, non-Aboriginal cohort with lung cancer and further repeated in cases of colon and head and neck cancers. In prostate cancer cases as the most frequently diagnosed cancer, frequent GP contact by Aboriginal men also had a positive effect on local stage diagnosis. There was no such effect among non-Aboriginal men. Without further research we can only speculate on the reasons behind this. However, it is plausible that more culturally appropriate care may influence screening participation and health protecting behaviours as noted earlier [51], even among a generally resistant clientele.

### Limitations and strengths

Our reporting format aligns with the NSW Ministry of Health and CINSW public reporting of major tumour streams [40] and key populations [54]. That consistency facilitates cross-referencing between operational reporting and research findings for decision-making and public engagement.

Our analysis contributes to a conceptual model [32] of the mitigating effects of frequent GP contact on the path from comorbidity to earlier stage of diagnosis among Aboriginal people with cancer. The data also indicate the disparate needs and experiences of older Aboriginal people with cancer [32].

## Implications and applications

Our study provides evidence of Aboriginal peoples’ experience of cancer, insofar as achievable with administrative records. There are the questions of whether this evidence will assist Aboriginal communities, health service providers, and community health care organisations to better address cancer. For example, will a diagrammatic representation of directional paths with accompanying quantification indicate the strength of influences on earlier cancer detection and care encourage narratives and storytelling in community settings?

## Further research

This is required to investigate:

- The nature of GP contacts that are related to earlier stage, including their timing, type, and organisational context. For example, are the GP consults conducted through private general practices, Aboriginal Community Controlled Organisations, or some combination of each.
- Different effects of GP contact by primary site as indicated in this study. Future analysis could explore the potential role of particular conditions, or condition combinations, with GP contact and earlier cancer detection.
- Evidence of the severity of comorbidity conditions’ impact on functional status, health related quality of life or health preferences. This would further increase understanding of the burden of comorbid conditions on the Aboriginal population.

## Conclusion

Older Aboriginal Australians experience more comorbid conditions, and along with socioeconomic disadvantage, this may reduce earlier cancer diagnosis. Frequent GP contact may partly counter these negative influences.

## Supplementary Information

Below is the link to the electronic supplementary material.

Supplementary file1 (DOCX 261 KB)

## Data Availability

The original data for this study were provided by the Australian Department of Health and the NSW Ministry of Health following approval by all relevant ethics committees. These data may be available to other researchers meeting the relevant data access and ethical requirements. Requests and enquiries on the data processing and analyses code for this article can be made to DB.

## References

[1] Australian Institute of Health, Welfare. (2019) Insights into vulnerabilities of Aboriginal, Torres Strait Islander people aged 50, over 2019.Canberra: AIHW

[2] HealthInfoNet AI. (2020) Summary of cancer among Aboriginal and Torres Strait Islander people Perth, W.A.: Australian Indigenous HealthInfoNet.

[3] Condon JR, Zhang X, Baade P, et al. (2014) Cancer survival for Aboriginal and Torres Strait Islander Australians: a national study of survival rates and excess mortality. Population Health Metrics. 12. 10.1186/1478-7954-12-1 PMC3909914 24479861

[4] Australian Institute of Health and Welfare. (2018) Cancer in Aboriginal & Torres Strait Islander people of Australia. Canberra: AIHW.

[5] Cunningham J, Rumbold A, Zhang X, Condon J (2008). Incidence, aetiology, and outcomes of cancer in Indigenous peoples in Australia. Lancet Oncol.

[6] Tervonen HE, Walton R, You H (2017). After accounting for competing causes of death and more advanced stage, do Aboriginal and Torres Strait Islander peoples with cancer still have worse survival? A population-based cohort study in New South Wales. BMC Cancer.

[7] Condon J, Armstrong B, Barnes A, Zhao Y (2005). Cancer incidence and survival for Indigenous Australians in the Northern Territory. Aust N Z J Public Health.

[8] Valery P, Coory M, Stirling J, Green A (2006). Cancer diagnosis, treatment, and survival in Indigenous and non-Indigenous Australians: a matched cohort study. Lancet.

[9] Banham D, Roder D, Keefe D (2017). Disparities in cancer stage at diagnosis and survival of Aboriginal and non-Aboriginal South Australians. Cancer Epidemiol.

[10] Chong A, Roder D (2010). Exploring differences in survival from cancer among Indigenous and non-Indigenous Australians: implications for health service delivery and research. Asian Pac J Cancer Prev.

[11] Diaz A, Whop LJ, Valery PC (2015). Cancer outcomes for Aboriginal and Torres Strait Islander Australians in rural and remote areas. Aust J Rural Health.

[12] Moore S, Green A, Bray F (2014). Survival disparities in Australia: an analysis of patterns of care and comorbidities among indigenous and non-indigenous cancer patients. BMC Cancer.

[13] Moore SP, O'Rourke PK, Mallitt K-A (2010). Cancer incidence and mortality in Indigenous Australians in Queensland, 1997–2006. Med J Aust.

[14] Sarfati D, Koczwara B, Jackson C. (2016) The impact of comorbidity on cancer and its treatment. CA: A Cancer Journal for Clinicians. 66: 338–50. 10.3322/caac.21342 26891458

[15] Sheppard AJ, Chiarelli AM, Marrett LD, Nishri ED, Trudeau ME (2011). Stage at Diagnosis and Comorbidity Influence Breast Cancer Survival in First Nations Women in Ontario. Canada. Cancer Epidemiology Biomarkers & Prevention..

[16] Gurney J, Stanley J, Sarfati D. (2020) The inequity of morbidity: Disparities in the prevalence of morbidity between ethnic groups in New Zealand. Journal of Comorbidity. 10: 2235042X20971168. 10.1177/2235042X20971168 PMC7658519 33224894

[17] Randall DA, Lujic S, Havard A, Eades SJ, Jorm L (2018). Multimorbidity among Aboriginal people in New South Wales contributes significantly to their higher mortality. Med J Aust.

[18] Roder D, Currow D (2009). Cancer in Aboriginal and Torres Strait Islander People of Australia. Asian Pac J Cancer Prev.

[19] Gurney J, Sarfati D, Stanley J (2015). The impact of patient comorbidity on cancer stage at diagnosis. Br J Cancer.

[20] Banham D, Brown A, Roder D (2018). Comorbidities contribute to the risk of cancer death among Aboriginal and non-Aboriginal South Australians: Analysis of a matched cohort study. Cancer Epidemiol.

[21] Banham D, Roder D, Thompson S, et al. (In review) Comorbidity among older Aboriginal and non-Aboriginal Australians with cancer in New South Wales. Journal of Multimorbidity and Comorbidity.

[22] Welfare AIoHa. (2021) Chronic condition multimorbidity. Canberra: AIHW.

[23] Goodwin JS, Samet JM, Key CR, Humble C, Kutvirt D, Hunt C (1986). Stage at diagnosis of cancer varies with the age of the patient. J Am Geriatr Soc.

[24] Lipscomb J, Fleming ST, Trentham-Dietz A (2016). What Predicts an Advanced-Stage Diagnosis of Breast Cancer? Sorting Out the Influence of Method of Detection, Access to Care, and Biologic Factors. Cancer Epidemiol Biomarkers Prev.

[25] Mazza D, Mitchell G (2017). Cancer, ageing, multimorbidity and primary care. Eur J Cancer Care.

[26] Fleming ST, Pursley HG, Newman B, Pavlov D, Chen K (2005). Comorbidity as a Predictor of Stage of Illness for Patients With Breast Cancer. Med Care.

[27] Koczwara B (2016). Cancer and Chronic Conditions: Addressing the Problem of Multimorbidity in Cancer Patients and Survivors.

[28] Australian Institute of Health and Welfare. (2014) Access to primary health care relative to need for Indigenous Australians. Canberra: AIHW.

[29] Johanson R, Hill P (2011). Indigenous health A role for private general practice. Aust Fam Physician.

[30] Maree P, Hughes R, Radford J, Stankovich J, Van Dam PJ (2020). Integrating patient complexity into health policy: a conceptual framework. Aust Health Rev.

[31] Purdie S, Creighton N, White KM (2019). Pathways to diagnosis of non-small cell lung cancer: a descriptive cohort study. NPJ Prim Care Respir Med..

[32] Geraci JM, Escalante CP, Freeman JL, Goodwin JS (2005). Comorbid disease and cancer: the need for more relevant conceptual models in health services research. Journal of clinical oncology : official journal of the American Society of Clinical Oncology..

[33] Australian Bureau of Statistics. (2014) Estimates and Projections, Aboriginal and Torres Strait Islander Australians: 2001 to 2026. Canberra: ABS.

[34] NSW Cancer Registry. Sydney: Cancer Institute NSW.

[35] NSW Ministry of Health. NSW Admitted Patient Data Collection (APDC). Sydney: Centre for Health Record Linkage (CHeReL).

[36] Australian Bureau of Statistics. (2013) Socio-Economic Indexes for Areas (SEIFA) - Technical Paper, 2011.

[37] Australian Bureau of Statistics. (2007) Australian Standard Geographical Classification (ASGC). Canberra: ABS.

[38] Independent Hospital Pricing Authority (IHPA). (2019) The International Statistical Classification of Diseases and Related Health Problems Australian Modification (ICD-10-AM). Sydney.

[39] Sax Institute. (2016) Secure Unified Research Environment (SURE).

[40] Cancer Institute NSW. (2019) Reporting for Better Cancer Outcomes (RBCO).

[41] Tervonen HE, Purdie S, Creighton N (2019). Using data linkage to enhance the reporting of cancer outcomes of Aboriginal and Torres Strait Islander people in NSW. Australia. BMC Med Res Methodol..

[42] Preen DB, Holman CDAJ, Spilsbury K, Semmens JB, Brameld KJ (2006). Length of comorbidity lookback period affected regression model performance of administrative health data. J Clin Epidemiol.

[43] Lu CY, Barratt JV, Agnes RE (2011). Charlson and Rx-Risk comorbidity indices were predictive of mortality in the Australian health care setting. J Clin Epidemiol.

[44] StataCorp. (2019) Stata Statistical Software: Release 16.0. College Station: StataCorp LP.

[45] Acock AC (2013) Discovering structural equation modeling using Stata. Stata Press Books, College Station.

[46] Hallgren KA, McCabe CJ, King KM, Atkins DC (2019). Beyond path diagrams: enhancing applied structural equation modeling research through data visualization. Addict Behav.

[47] Hosmer D, Lemeshow S (2000). Applied logistic regression.

[48] Tu Y-K, Greenwood DC (2012). Modern methods for epidemiology.

[49] Altman DG, Royston P (2006). The cost of dichotomising continuous variables. BMJ.

[50] Shahid S, Teng T, Bessarab D, Aoun S, Baxi S, Thompson S (2016). Factors contributing to delayed diagnosis of cancer among Aboriginal people in Australia: a qualitative study. BMJ open.

[51] Harfield SG, Davy C, McArthur A, Munn Z, Brown A, Brown N (2018). Characteristics of Indigenous primary health care service delivery models: a systematic scoping review. Glob Health.

[52] Australian Bureau of Statistics. (2020) Patient Experiences in Australia Summary of Findings. Canberra: ABS.

[53] Condon JR, Cunningham J, Barnes T, Armstrong BK, Selva-Nayagam S (2006). Cancer diagnosis and treatment in the Northern Territory: assessing health service performance for indigenous Australians. Intern Med J.

[54] Cancer Institute NSW (2020). Reporting for better cancer outcomes (RBCO): aboriginal people in NSW.

