# Supplementary material for: The effect of general practice contact on cancer stage at diagnosis in Aboriginal and non-Aboriginal residents of New South Wales
Source: Cancer Causes Control. 2023 Jun 17;34(10):909–26. doi: 10.1007/s10552-023-01727-6 (PMC10460337; doi:10.1007/s10552-023-01727-6)
Supplement: Supplementary file 1 — Supplementary file1 (DOCX 261 KB) [file 10552_2023_1727_MOESM1_ESM.docx]

**Supplementary material**

**
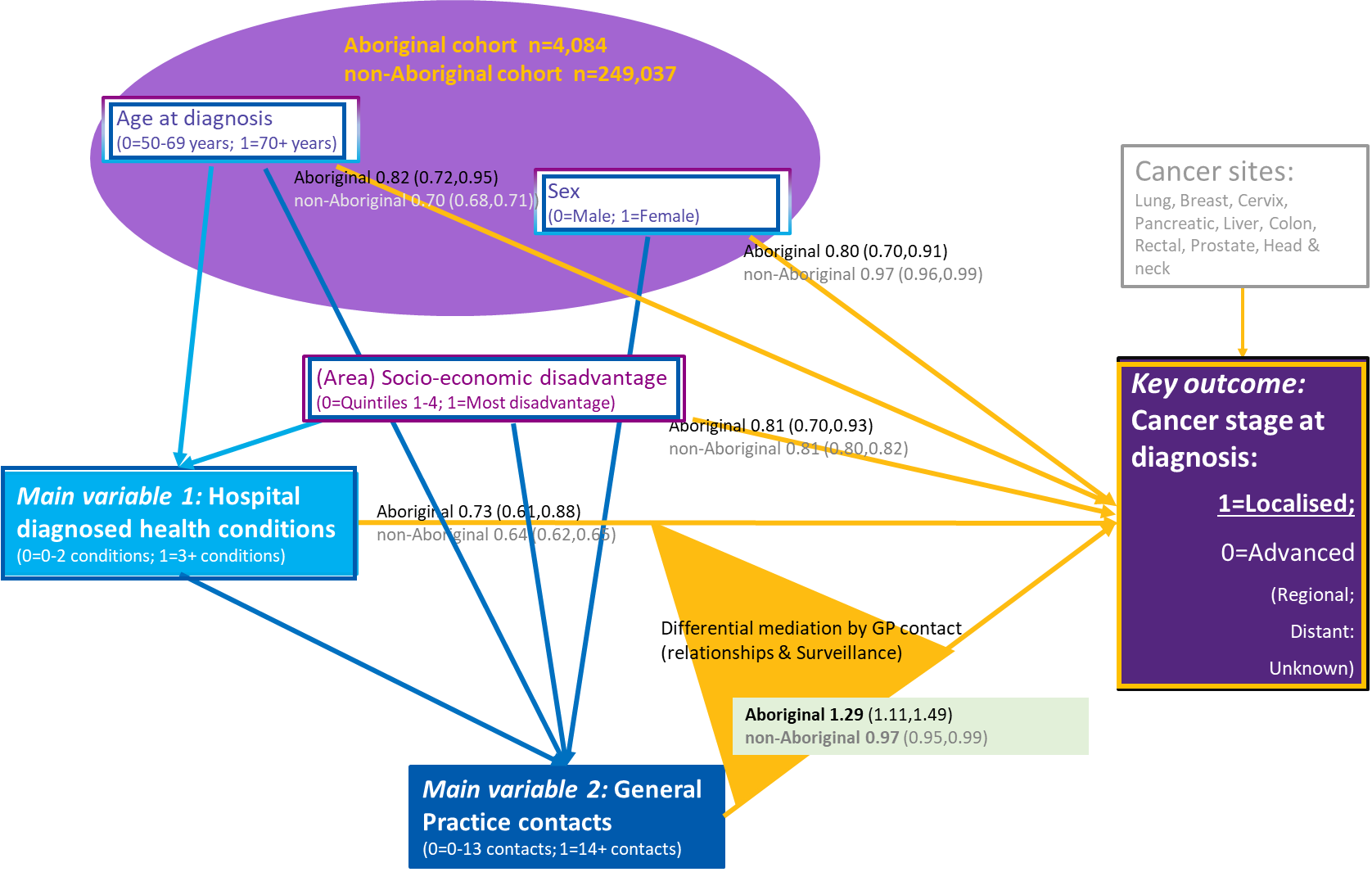
**

**Supplementary Figure S1: Structural Equation Modelled multivariable associations of comorbidity, General Practice visits and local stage at diagnosis outcomes among Aboriginal and non-Aboriginal cohorts aged 50 years or more at first invasive cancer diagnosis in New South Wales, July 2003 to December 2016**

**Supplementary Table S1: Sensitivity of Structural Equation Modelled multivariable associations of comorbidity, General Practice visits and local stage at diagnosis outcomes among Aboriginal and non-Aboriginal cohorts aged 50 years or more at first invasive cancer diagnosis in New South Wales, July 2003 to December 2016**

|  | Reduced cohorts (with Border LHDs excluded) | | | | | |  | Full cohorts (Border LHDs included in Outcome 1 model) | | | | | |
| --- | --- | --- | --- | --- | --- | --- | --- | --- | --- | --- | --- | --- | --- |
|  | **Aboriginal cohort** | | | **non-Aboriginal cohort** | | |  | **Aboriginal cohort** | | | **non-Aboriginal cohort** | | |
|  | Odds Ratio (adjusted) | L95%CI | U95%CI | Odds Ratio (adjusted) | L95%CI | U95%CI |  | Odds Ratio (adjusted) | L95%CI | U95%CI | Odds Ratio (adjusted) | L95%CI | U95%CI |
| **Main variable 1: 3 or more comorbid conditions** |  |  |  |  |  |  |  |  |  |  |  |  |  |
| Age group at diagnosis |  |  |  |  |  |  |  |  |  |  |  |  |  |
| 50 to 69 years | 1.00 | Reference | | 1.00 | Reference | |  | 1.00 | Reference | | 1.00 | Reference | |
| 70 or more years | 1.80 | 1.50 | 2.15 | 3.04 | 2.95 | 3.13 |  | 1.81 | 1.53 | 2.13 | 3.05 | 2.97 | 3.13 |
| Area level Index of Relative Socio-economic Disadvantage (IRSD) | | |  |  |  |  |  |  |  |  |  |  |  |
| Least disadvantage Quintiles 1 to 4 | 1.00 | Reference | | 1.00 | Reference | |  | 1.00 | Reference | | 1.00 | Reference | |
| Most disadvantage Quintile 5 | 1.45 | 1.19 | 1.77 | 1.39 | 1.35 | 1.43 |  | 1.56 | 1.29 | 1.88 | 1.41 | 1.37 | 1.44 |
| Reside near state border |  |  |  |  |  |  |  |  |  |  |  |  |  |
| No |  |  |  |  |  |  |  | 1.00 | Reference | | 1.00 | Reference | |
| Yes |  |  |  |  |  |  |  | 0.76 | 0.61 | 0.95 | 0.68 | 0.65 | 0.70 |
| **Main variable 2: 14 or more GP consults** |  |  |  |  |  |  |  |  |  |  |  |  |  |
| Comorbid conditions (Elixhauser) |  |  |  |  |  |  |  |  |  |  |  |  |  |
| 0 to 2 conditions | 1.00 | Reference | | 1.00 | Reference | |  | 1.00 | Reference | | 1.00 | Reference | |
| 3 or more conditions | 2.02 | 1.69 | 2.42 | 2.63 | 2.56 | 2.70 |  | 2.10 | 1.78 | 2.47 | 2.68 | 2.61 | 2.75 |
| **Key outcome: Local stage at diagnosis** |  |  |  |  |  |  |  |  |  |  |  |  |  |
| Age group at diagnosis |  |  |  |  |  |  |  |  |  |  |  |  |  |
| 50 to 69 years | 1.00 | Reference | | 1.00 | Reference | |  | 1.00 | Reference | | 1.00 | Reference | |
| 70 or more years | 0.83 | 0.71 | 0.98 | 0.70 | 0.69 | 0.71 |  | 0.84 | 0.73 | 0.97 | 0.70 | 0.69 | 0.71 |
| Sex |  |  |  |  |  |  |  |  |  |  |  |  |  |
| Male | 1.00 | Reference | | 1.00 | Reference | |  | 1.00 | Reference | | 1.00 | Reference | |
| Female | 0.81 | 0.70 | 0.94 | 0.96 | 0.94 | 0.98 |  | 0.80 | 0.70 | 0.91 | 0.97 | 0.96 | 0.99 |
| Area level Index of Relative Socio-economic Disadvantage (IRSD) | | |  |  |  |  |  |  |  |  |  |  |  |
| Least disadvantage Quintiles 1 to 4 | 1.00 | Reference | | 1.00 | Reference | |  | 1.00 | Reference | | 1.00 | Reference | |
| Most disadvantage Quintile 5 | 0.83 | 0.71 | 0.96 | 0.80 | 0.78 | 0.81 |  | 0.80 | 0.69 | 0.92 | 0.81 | 0.80 | 0.82 |
| Comorbid conditions (Elixhauser) |  |  |  |  |  |  |  |  |  |  |  |  |  |
| 0 to 2 conditions | 1.00 | Reference | | 1.00 | Reference | |  | 1.00 | Reference | | 1.00 | Reference | |
| 3 or more conditions | 0.74 | 0.61 | 0.90 | 0.63 | 0.61 | 0.64 |  | 0.75 | 0.63 | 0.89 | 0.64 | 0.62 | 0.66 |
| GP consults |  |  |  |  |  |  |  |  |  |  |  |  |  |
| 0 to 13 consults | 1.00 | Reference | | 1.00 | Reference | |  | 1.00 | Reference | | 1.00 | Reference | |
| 14 or more consults | 1.26 | 1.08 | 1.48 | 0.95 | 0.93 | 0.97 |  | 1.22 | 1.06 | 1.40 | 0.94 | 0.93 | 0.96 |

**Supplementary Table S2: Structural Equation Modelled multivariable associations of comorbidity, General Practice visits and local stage at diagnosis outcomes among Aboriginal and non-Aboriginal cohorts stratified by primary cancer site**

|  | **a. Lung** |  |  |  |  |  |  |  |
| --- | --- | --- | --- | --- | --- | --- | --- | --- |
|  | Aboriginal cohort | | | | non-Aboriginal cohort | | | |
|  | Odds Ratio (adjusted) | Lower 95% Confidence Interval | Upper 95% Confidence Interval | p | Odds Ratio (adjusted) | Lower 95% Confidence Interval | Upper 95% Confidence Interval | p |
| Main variable 1: 3 or more comorbid conditions | |  |  |  |  |  |  |  |
| Age group at diagnosis |  |  |  |  |  |  |  |  |
| 50 to 69 years | 1.00 | Reference | |  | 1.00 | Reference | |  |
| 70 or more years | 1.83 | 1.36 | 2.47 | 0.000 | 1.86 | 1.76 | 1.96 | 0.000 |
| Area level Index of Relative Socio-economic Disadvantage (IRSD) | | |  |  |  |  |  |  |
| Least disadvantage Quintiles 1 to 4 | 1.00 | Reference | |  | 1.00 | Reference | |  |
| Most disadvantage Quintile 5 | 0.95 | 0.67 | 1.36 | 0.790 | 1.18 | 1.13 | 1.25 | 0.000 |
| Main variable 2: 14 or more GP consults |  |  |  |  |  |  |  |  |
| Age group at diagnosis |  |  |  |  |  |  |  |  |
| 50 to 69 years | 1.00 | Reference | |  | 1.00 | Reference | |  |
| 70 or more years | 1.87 | 1.41 | 2.47 | 0.000 | 1.84 | 1.76 | 1.92 | 0.000 |
| Sex |  |  |  |  |  |  |  |  |
| Male | 1.00 | Reference | |  | 1.00 | Reference | |  |
| Female | 1.33 | 1.01 | 1.76 | 0.040 | 1.19 | 1.14 | 1.24 | 0.000 |
| Area level Index of Relative Socio-economic Disadvantage (IRSD) | | |  |  |  |  |  |  |
| Least disadvantage Quintiles 1 to 4 | 1.00 | Reference | |  | 1.00 | Reference | |  |
| Most disadvantage Quintile 5 | 1.03 | 0.74 | 1.44 | 0.844 | 0.99 | 0.95 | 1.03 | 0.600 |
| Comorbid conditions (Elixhauser) |  |  |  |  |  |  |  |  |
| 0 to 2 conditions | 1.00 | Reference | |  | 1.00 | Reference | |  |
| 3 or more conditions | 1.74 | 1.27 | 2.37 | 0.001 | 1.94 | 1.84 | 2.05 | 0.000 |
| Key outcome: Local-stage at diagnosis |  |  |  |  |  |  |  |  |
| Age group at diagnosis |  |  |  |  |  |  |  |  |
| 50 to 69 years | 1.00 | Reference | |  | 1.00 | Reference | |  |
| 70 or more years | 1.08 | 0.77 | 1.52 | 0.659 | 1.03 | 0.98 | 1.09 | 0.227 |
| Sex |  |  |  |  |  |  |  |  |
| Male | 1.00 | Reference | |  | 1.00 | Reference | |  |
| Female | 0.91 | 0.65 | 1.26 | 0.56 | 1.15 | 1.09 | 1.21 | 0.00 |
| Area level Index of Relative Socio-economic Disadvantage (IRSD) | | |  |  |  |  |  |  |
| Least disadvantage Quintiles 1 to 4 | 1.00 | Reference | |  | 1.00 | Reference | |  |
| Most disadvantage Quintile 5 | 1.25 | 0.82 | 1.90 | 0.000 | 0.95 | 0.91 | 1.00 | 0.07 |
| Comorbid conditions (Elixhauser) |  |  |  |  |  |  |  |  |
| 0 to 2 conditions | 1.00 | Reference | |  | 1.00 | Reference | |  |
| 3 or more conditions | 1.38 | 0.95 | 1.99 | 0.091 | 0.96 | 0.90 | 1.03 | 0.254 |
| GP consults |  |  |  |  |  |  |  |  |
| 0 to 13 consults | 1.00 | Reference | |  | 1.00 | Reference | |  |
| 14 or more consults | 1.58 | 1.12 | 2.22 | 0.010 | 1.26 | 1.19 | 1.33 | 0.000 |

|  | **b. Breast (female)** | |  |  |  |  |  |  |
| --- | --- | --- | --- | --- | --- | --- | --- | --- |
|  | **Aboriginal cohort** | | | | **non-Aboriginal cohort** | | | |
|  | Odds Ratio (adjusted) | Lower 95% Confidence Interval | Upper 95% Confidence Interval | p | Odds Ratio (adjusted) | Lower 95% Confidence Interval | Upper 95% Confidence Interval | p |
| **Main variable 1: 3 or more comorbid conditions** | |  |  |  |  |  |  |  |
| Age group at diagnosis |  |  |  |  |  |  |  |  |
| 50 to 69 years | 1.00 | Reference | |  | 1.00 | Reference | |  |
| 70 or more years | 1.64 | 0.87 | 3.08 | 0.126 | 4.79 | 4.30 | 5.33 | 0.000 |
| Area level Index of Relative Socio-economic Disadvantage (IRSD) | | |  |  |  |  |  |  |
| Least disadvantage Quintiles 1 to 4 | 1.00 | Reference | |  | 1.00 | Reference | |  |
| Most disadvantage Quintile 5 | 2.87 | 1.33 | 6.19 | 0.007 | 1.36 | 1.23 | 1.50 | 0.000 |
| **Main variable 2: 14 or more GP consults** |  |  |  |  |  |  |  |  |
| Age group at diagnosis |  |  |  |  |  |  |  |  |
| 50 to 69 years | 1.00 | Reference | |  | 1.00 | Reference | |  |
| 70 or more years | 2.03 | 1.34 | 3.08 | 0.001 | 2.70 | 2.57 | 2.83 | 0.000 |
| Sex |  |  |  |  |  |  |  |  |
| Male |  |  | |  |  |  | |  |
| Female |  |  |  |  |  |  |  |  |
| Area level Index of Relative Socio-economic Disadvantage (IRSD) | | |  |  |  |  |  |  |
| Least disadvantage Quintiles 1 to 4 | 1.00 | Reference | |  | 1.00 | Reference | |  |
| Most disadvantage Quintile 5 | 1.32 | 0.88 | 1.98 | 0.179 | 1.20 | 1.15 | 1.26 | 0.000 |
| Comorbid conditions (Elixhauser) |  |  |  |  |  |  |  |  |
| 0 to 2 conditions | 1.00 | Reference | |  | 1.00 | Reference | |  |
| 3 or more conditions | 3.85 | 2.16 | 6.86 | 0.000 | 3.60 | 3.25 | 3.99 | 0.000 |
| **Key outcome: Local-stage at diagnosis** |  |  |  |  |  |  |  |  |
| Age group at diagnosis |  |  |  |  |  |  |  |  |
| 50 to 69 years | 1.00 | Reference | |  | 1.00 | Reference | |  |
| 70 or more years | 0.56 | 0.38 | 0.82 | 0.003 | 0.76 | 0.73 | 0.79 | 0.000 |
| Sex |  |  |  |  |  |  |  |  |
| Male |  |  | |  |  |  | |  |
| Female |  |  |  |  |  |  |  |  |
| Area level Index of Relative Socio-economic Disadvantage (IRSD) | | |  |  |  |  |  |  |
| Least disadvantage Quintiles 1 to 4 | 1.00 | Reference | |  | 1.00 | Reference | |  |
| Most disadvantage Quintile 5 | 0.84 | 0.61 | 1.16 | 0.126 | 0.93 | 0.90 | 0.97 | 0.00 |
| Comorbid conditions (Elixhauser) |  |  |  |  |  |  |  |  |
| 0 to 2 conditions | 1.00 | Reference | |  | 1.00 | Reference | |  |
| 3 or more conditions | 0.65 | 0.36 | 1.17 | 0.148 | 0.61 | 0.55 | 0.68 | 0.000 |
| GP consults |  |  |  |  |  |  |  |  |
| 0 to 13 consults | 1.00 | Reference | |  | 1.00 | Reference | |  |
| 14 or more consults | 1.35 | 0.93 | 1.94 | 0.112 | 1.06 | 1.01 | 1.11 | 0.026 |

|  | **c. Cervix** |  |  |  |  |  |  |  |
| --- | --- | --- | --- | --- | --- | --- | --- | --- |
|  | **Aboriginal cohort** | | | | **non-Aboriginal cohort** | | | |
|  | Odds Ratio (adjusted) | Lower 95% Confidence Interval | Upper 95% Confidence Interval | p | Odds Ratio (adjusted) | Lower 95% Confidence Interval | Upper 95% Confidence Interval | p |
| **Main variable 1: 3 or more comorbid conditions** | |  |  |  |  |  |  |  |
| Age group at diagnosis |  |  |  |  |  |  |  |  |
| 50 to 69 years | 1.00 | Reference | |  | 1.00 | Reference | |  |
| 70 or more years | 0.96 | 0.17 | 5.39 | 0.961 | 2.89 | 2.08 | 4.02 | 0.000 |
| Area level Index of Relative Socio-economic Disadvantage (IRSD) | | |  |  |  |  |  |  |
| Least disadvantage Quintiles 1 to 4 | 1.00 | Reference | |  | 1.00 | Reference | |  |
| Most disadvantage Quintile 5 | 1.89 | 0.20 | 17.45 | 0.575 | 1.63 | 1.16 | 2.27 | 0.005 |
| **Main variable 2: 14 or more GP consults** |  |  |  |  |  |  |  |  |
| Age group at diagnosis |  |  |  |  |  |  |  |  |
| 50 to 69 years | 1.00 | Reference | |  | 1.00 | Reference | |  |
| 70 or more years | 2.44 | 0.48 | 12.52 | 0.284 | 2.44 | 1.87 | 3.20 | 0.000 |
| Sex |  |  |  |  |  |  |  |  |
| Male |  |  | |  |  |  | |  |
| Female |  |  |  |  |  |  |  |  |
| Area level Index of Relative Socio-economic Disadvantage (IRSD) | | |  |  |  |  |  |  |
| Least disadvantage Quintiles 1 to 4 | 1.00 | Reference | |  | 1.00 | Reference | |  |
| Most disadvantage Quintile 5 | 0.27 | 0.05 | 1.50 | 0.134 | 1.30 | 0.99 | 1.70 | 0.057 |
| Comorbid conditions (Elixhauser) |  |  |  |  |  |  |  |  |
| 0 to 2 conditions | 1.00 | Reference | |  | 1.00 | Reference | |  |
| 3 or more conditions | 0.78 | 0.14 | 4.44 | 0.784 | 1.69 | 1.17 | 2.45 | 0.005 |
| **Key outcome: Local-stage at diagnosis** |  |  |  |  |  |  |  |  |
| Age group at diagnosis |  |  |  |  |  |  |  |  |
| 50 to 69 years | 1.00 | Reference | |  | 1.00 | Reference | |  |
| 70 or more years | 0.15 | 0.01 | 2.00 | 0.150 | 0.69 | 0.55 | 0.87 | 0.002 |
| Sex |  |  |  |  |  |  |  |  |
| Male |  |  | |  |  |  | |  |
| Female |  |  |  |  |  |  |  |  |
| Area level Index of Relative Socio-economic Disadvantage (IRSD) | | |  |  |  |  |  |  |
| Least disadvantage Quintiles 1 to 4 | 1.00 | Reference | |  | 1.00 | Reference | |  |
| Most disadvantage Quintile 5 | 0.74 | 0.08 | 6.54 | 0.961 | 0.76 | 0.61 | 0.94 | 0.01 |
| Comorbid conditions (Elixhauser) |  |  |  |  |  |  |  |  |
| 0 to 2 conditions | 1.00 | Reference | |  | 1.00 | Reference | |  |
| 3 or more conditions | 2.47 | 0.45 | 13.67 | 0.301 | 0.58 | 0.39 | 0.85 | 0.006 |
| GP consults |  |  |  |  |  |  |  |  |
| 0 to 13 consults | 1.00 | Reference | |  | 1.00 | Reference | |  |
| 14 or more consults | 17.34 | 2.64 | 113.91 | 0.003 | 1.24 | 0.93 | 1.64 | 0.142 |

|  | **d. Pancreatic** | |  |  |  |  |  |  |
| --- | --- | --- | --- | --- | --- | --- | --- | --- |
|  | **Aboriginal cohort** | | | | **non-Aboriginal cohort** | | | |
|  | Odds Ratio (adjusted) | Lower 95% Confidence Interval | Upper 95% Confidence Interval | p | Odds Ratio (adjusted) | Lower 95% Confidence Interval | Upper 95% Confidence Interval | p |
| **Main variable 1: 3 or more comorbid conditions** | |  |  |  |  |  |  |  |
| Age group at diagnosis |  |  |  |  |  |  |  |  |
| 50 to 69 years | 1.00 | Reference | |  | 1.00 | Reference | |  |
| 70 or more years | 1.47 | 0.78 | 2.74 | 0.232 | 1.62 | 1.47 | 1.79 | 0.000 |
| Area level Index of Relative Socio-economic Disadvantage (IRSD) | | |  |  |  |  |  |  |
| Least disadvantage Quintiles 1 to 4 | 1.00 | Reference | |  | 1.00 | Reference | |  |
| Most disadvantage Quintile 5 | 1.46 | 0.70 | 3.05 | 0.308 | 1.10 | 1.00 | 1.20 | 0.048 |
| **Main variable 2: 14 or more GP consults** |  |  |  |  |  |  |  |  |
| Age group at diagnosis |  |  |  |  |  |  |  |  |
| 50 to 69 years | 1.00 | Reference | |  | 1.00 | Reference | |  |
| 70 or more years | 2.02 | 1.10 | 3.72 | 0.024 | 1.59 | 1.47 | 1.73 | 0.000 |
| Sex |  |  |  |  |  |  |  |  |
| Male | 1.00 | Reference | |  | 1.00 | Reference | |  |
| Female | 1.47 | 0.80 | 2.71 | 0.218 | 1.07 | 0.99 | 1.16 | 0.107 |
| Area level Index of Relative Socio-economic Disadvantage (IRSD) | | |  |  |  |  |  |  |
| Least disadvantage Quintiles 1 to 4 | 1.00 | Reference | |  | 1.00 | Reference | |  |
| Most disadvantage Quintile 5 | 1.05 | 0.52 | 2.10 | 0.893 | 1.04 | 0.96 | 1.13 | 0.321 |
| Comorbid conditions (Elixhauser) |  |  |  |  |  |  |  |  |
| 0 to 2 conditions | 1.00 | Reference | |  | 1.00 | Reference | |  |
| 3 or more conditions | 1.47 | 0.77 | 2.78 | 0.241 | 1.95 | 1.78 | 2.14 | 0.000 |
| **Key outcome: Local-stage at diagnosis** |  |  |  |  |  |  |  |  |
| Age group at diagnosis |  |  |  |  |  |  |  |  |
| 50 to 69 years | 1.00 | Reference | |  | 1.00 | Reference | |  |
| 70 or more years | 1.26 | 0.53 | 3.00 | 0.608 | 1.28 | 1.14 | 1.44 | 0.000 |
| Sex |  |  |  |  |  |  |  |  |
| Male | 1.00 | Reference | |  | 1.00 | Reference | |  |
| Female | 1.76 | 0.71 | 4.37 | 0.22 | 1.11 | 1.00 | 1.23 | 0.06 |
| Area level Index of Relative Socio-economic Disadvantage (IRSD) | | |  |  |  |  |  |  |
| Least disadvantage Quintiles 1 to 4 | 1.00 | Reference | |  | 1.00 | Reference | |  |
| Most disadvantage Quintile 5 | 1.21 | 0.44 | 3.30 | 0.232 | 0.97 | 0.87 | 1.08 | 0.55 |
| Comorbid conditions (Elixhauser) |  |  |  |  |  |  |  |  |
| 0 to 2 conditions | 1.00 | Reference | |  | 1.00 | Reference | |  |
| 3 or more conditions | 1.38 | 0.57 | 3.33 | 0.476 | 1.21 | 1.07 | 1.37 | 0.002 |
| GP consults |  |  |  |  |  |  |  |  |
| 0 to 13 consults | 1.00 | Reference | |  | 1.00 | Reference | |  |
| 14 or more consults | 1.48 | 0.62 | 3.52 | 0.375 | 1.08 | 0.97 | 1.20 | 0.163 |

|  | **e. Liver** |  |  |  |  |  |  |  |
| --- | --- | --- | --- | --- | --- | --- | --- | --- |
|  | **Aboriginal cohort** | | | | **non-Aboriginal cohort** | | | |
|  | Odds Ratio (adjusted) | Lower 95% Confidence Interval | Upper 95% Confidence Interval | p | Odds Ratio (adjusted) | Lower 95% Confidence Interval | Upper 95% Confidence Interval | p |
| **Main variable 1: 3 or more comorbid conditions** | |  |  |  |  |  |  |  |
| Age group at diagnosis |  |  |  |  |  |  |  |  |
| 50 to 69 years | 1.00 | Reference | |  | 1.00 | Reference | |  |
| 70 or more years | 0.86 | 0.43 | 1.71 | 0.673 | 1.12 | 1.01 | 1.25 | 0.031 |
| Area level Index of Relative Socio-economic Disadvantage (IRSD) | | |  |  |  |  |  |  |
| Least disadvantage Quintiles 1 to 4 | 1.00 | Reference | |  | 1.00 | Reference | |  |
| Most disadvantage Quintile 5 | 0.92 | 0.49 | 1.75 | 0.808 | 1.04 | 0.93 | 1.15 | 0.505 |
| **Main variable 2: 14 or more GP consults** |  |  |  |  |  |  |  |  |
| Age group at diagnosis |  |  |  |  |  |  |  |  |
| 50 to 69 years | 1.00 | Reference | |  | 1.00 | Reference | |  |
| 70 or more years | 2.16 | 1.07 | 4.35 | 0.031 | 1.64 | 1.47 | 1.82 | 0.000 |
| Sex |  |  |  |  |  |  |  |  |
| Male | 1.00 | Reference | |  | 1.00 | Reference | |  |
| Female | 1.46 | 0.73 | 2.93 | 0.286 | 1.06 | 0.95 | 1.19 | 0.285 |
| Area level Index of Relative Socio-economic Disadvantage (IRSD) | | |  |  |  |  |  |  |
| Least disadvantage Quintiles 1 to 4 | 1.00 | Reference | |  | 1.00 | Reference | |  |
| Most disadvantage Quintile 5 | 1.45 | 0.73 | 2.87 | 0.286 | 1.02 | 0.92 | 1.13 | 0.773 |
| Comorbid conditions (Elixhauser) |  |  |  |  |  |  |  |  |
| 0 to 2 conditions | 1.00 | Reference | |  | 1.00 | Reference | |  |
| 3 or more conditions | 1.14 | 0.63 | 2.07 | 0.659 | 1.80 | 1.61 | 2.00 | 0.000 |
| **Key outcome: Local-stage at diagnosis** |  |  |  |  |  |  |  |  |
| Age group at diagnosis |  |  |  |  |  |  |  |  |
| 50 to 69 years | 1.00 | Reference | |  | 1.00 | Reference | |  |
| 70 or more years | 1.18 | 0.58 | 2.41 | 0.653 | 0.69 | 0.62 | 0.76 | 0.000 |
| Sex |  |  |  |  |  |  |  |  |
| Male | 1.00 | Reference | |  | 1.00 | Reference | |  |
| Female | 0.61 | 0.30 | 1.26 | 0.18 | 0.90 | 0.80 | 1.01 | 0.06 |
| Area level Index of Relative Socio-economic Disadvantage (IRSD) | | |  |  |  |  |  |  |
| Least disadvantage Quintiles 1 to 4 | 1.00 | Reference | |  | 1.00 | Reference | |  |
| Most disadvantage Quintile 5 | 0.87 | 0.45 | 1.69 | 0.673 | 0.85 | 0.76 | 0.94 | 0.00 |
| Comorbid conditions (Elixhauser) |  |  |  |  |  |  |  |  |
| 0 to 2 conditions | 1.00 | Reference | |  | 1.00 | Reference | |  |
| 3 or more conditions | 1.36 | 0.76 | 2.44 | 0.308 | 1.28 | 1.15 | 1.42 | 0.000 |
| GP consults |  |  |  |  |  |  |  |  |
| 0 to 13 consults | 1.00 | Reference | |  | 1.00 | Reference | |  |
| 14 or more consults | 0.92 | 0.50 | 1.69 | 0.784 | 1.25 | 1.13 | 1.39 | 0.000 |

|  | **f. Colon** |  |  |  |  |  |  |  |
| --- | --- | --- | --- | --- | --- | --- | --- | --- |
|  | **Aboriginal cohort** | | | | **non-Aboriginal cohort** | | | |
|  | Odds Ratio (adjusted) | Lower 95% Confidence Interval | Upper 95% Confidence Interval | p | Odds Ratio (adjusted) | Lower 95% Confidence Interval | Upper 95% Confidence Interval | p |
| **Main variable 1: 3 or more comorbid conditions** | |  |  |  |  |  |  |  |
| Age group at diagnosis |  |  |  |  |  |  |  |  |
| 50 to 69 years | 1.00 | Reference | |  | 1.00 | Reference | |  |
| 70 or more years | 2.14 | 1.40 | 3.27 | 0.000 | 2.94 | 2.76 | 3.13 | 0.000 |
| Area level Index of Relative Socio-economic Disadvantage (IRSD) | | |  |  |  |  |  |  |
| Least disadvantage Quintiles 1 to 4 | 1.00 | Reference | |  | 1.00 | Reference | |  |
| Most disadvantage Quintile 5 | 1.66 | 1.03 | 2.68 | 0.039 | 1.20 | 1.14 | 1.26 | 0.000 |
| **Main variable 2: 14 or more GP consults** |  |  |  |  |  |  |  |  |
| Age group at diagnosis |  |  |  |  |  |  |  |  |
| 50 to 69 years | 1.00 | Reference | |  | 1.00 | Reference | |  |
| 70 or more years | 1.64 | 1.09 | 2.49 | 0.019 | 2.25 | 2.13 | 2.38 | 0.000 |
| Sex |  |  |  |  |  |  |  |  |
| Male | 1.00 | Reference | |  | 1.00 | Reference | |  |
| Female | 1.32 | 0.87 | 2.00 | 0.187 | 1.13 | 1.08 | 1.19 | 0.000 |
| Area level Index of Relative Socio-economic Disadvantage (IRSD) | | |  |  |  |  |  |  |
| Least disadvantage Quintiles 1 to 4 | 1.00 | Reference | |  | 1.00 | Reference | |  |
| Most disadvantage Quintile 5 | 1.01 | 0.65 | 1.58 | 0.954 | 0.99 | 0.94 | 1.04 | 0.715 |
| Comorbid conditions (Elixhauser) |  |  |  |  |  |  |  |  |
| 0 to 2 conditions | 1.00 | Reference | |  | 1.00 | Reference | |  |
| 3 or more conditions | 2.23 | 1.43 | 3.48 | 0.000 | 2.34 | 2.22 | 2.48 | 0.000 |
| **Key outcome: Local-stage at diagnosis** |  |  |  |  |  |  |  |  |
| Age group at diagnosis |  |  |  |  |  |  |  |  |
| 50 to 69 years | 1.00 | Reference | |  | 1.00 | Reference | |  |
| 70 or more years | 1.08 | 0.70 | 1.66 | 0.743 | 0.99 | 0.95 | 1.04 | 0.774 |
| Sex |  |  |  |  |  |  |  |  |
| Male | 1.00 | Reference | |  | 1.00 | Reference | |  |
| Female | 0.77 | 0.50 | 1.18 | 0.225 | 1.00 | 0.96 | 1.05 | 0.870 |
| Area level Index of Relative Socio-economic Disadvantage (IRSD) | | |  |  |  |  |  |  |
| Least disadvantage Quintiles 1 to 4 | 1.00 | Reference | |  | 1.00 | Reference | |  |
| Most disadvantage Quintile 5 | 1.12 | 0.70 | 1.77 | 0.000 | 0.92 | 0.88 | 0.96 | 0.000 |
| Comorbid conditions (Elixhauser) |  |  |  |  |  |  |  |  |
| 0 to 2 conditions | 1.00 | Reference | |  | 1.00 | Reference | |  |
| 3 or more conditions | 1.00 | 0.62 | 1.63 | 0.996 | 0.83 | 0.78 | 0.88 | 0.000 |
| GP consults |  |  |  |  |  |  |  |  |
| 0 to 13 consults | 1.00 | Reference | |  | 1.00 | Reference | |  |
| 14 or more consults | 1.48 | 0.94 | 2.32 | 0.092 | 1.16 | 1.10 | 1.22 | 0.000 |

|  | **g. Rectal** |  |  |  |  |  |  |  |
| --- | --- | --- | --- | --- | --- | --- | --- | --- |
|  | **Aboriginal cohort** | | | | **non-Aboriginal cohort** | | | |
|  | Odds Ratio (adjusted) | Lower 95% Confidence Interval | Upper 95% Confidence Interval | p | Odds Ratio (adjusted) | Lower 95% Confidence Interval | Upper 95% Confidence Interval | p |
| **Main variable 1: 3 or more comorbid conditions** | |  |  |  |  |  |  |  |
| Age group at diagnosis |  |  |  |  |  |  |  |  |
| 50 to 69 years | 1.00 | Reference | |  | 1.00 | Reference | |  |
| 70 or more years | 1.68 | 0.90 | 3.13 | 0.101 | 2.86 | 2.60 | 3.16 | 0.000 |
| Area level Index of Relative Socio-economic Disadvantage (IRSD) | | |  |  |  |  |  |  |
| Least disadvantage Quintiles 1 to 4 | 1.00 | Reference | |  | 1.00 | Reference | |  |
| Most disadvantage Quintile 5 | 1.21 | 0.63 | 2.33 | 0.575 | 1.22 | 1.11 | 1.34 | 0.000 |
| **Main variable 2: 14 or more GP consults** |  |  |  |  |  |  |  |  |
| Age group at diagnosis |  |  |  |  |  |  |  |  |
| 50 to 69 years | 1.00 | Reference | |  | 1.00 | Reference | |  |
| 70 or more years | 2.37 | 1.32 | 4.25 | 0.004 | 2.40 | 2.23 | 2.59 | 0.000 |
| Sex |  |  |  |  |  |  |  |  |
| Male | 1.00 | Reference | |  | 1.00 | Reference | |  |
| Female | 1.36 | 0.76 | 2.42 | 0.296 | 1.20 | 1.12 | 1.29 | 0.000 |
| Area level Index of Relative Socio-economic Disadvantage (IRSD) | | |  |  |  |  |  |  |
| Least disadvantage Quintiles 1 to 4 | 1.00 | Reference | |  | 1.00 | Reference | |  |
| Most disadvantage Quintile 5 | 1.12 | 0.61 | 2.07 | 0.711 | 1.07 | 1.00 | 1.15 | 0.061 |
| Comorbid conditions (Elixhauser) |  |  |  |  |  |  |  |  |
| 0 to 2 conditions | 1.00 | Reference | |  | 1.00 | Reference | |  |
| 3 or more conditions | 3.66 | 1.94 | 6.92 | 0.000 | 2.68 | 2.43 | 2.95 | 0.000 |
| **Key outcome: Local-stage at diagnosis** |  |  |  |  |  |  |  |  |
| Age group at diagnosis |  |  |  |  |  |  |  |  |
| 50 to 69 years | 1.00 | Reference | |  | 1.00 | Reference | |  |
| 70 or more years | 1.15 | 0.62 | 2.10 | 0.661 | 1.02 | 0.96 | 1.09 | 0.486 |
| Sex |  |  |  |  |  |  |  |  |
| Male | 1.00 | Reference | |  | 1.00 | Reference | |  |
| Female | 0.94 | 0.53 | 1.66 | 0.821 | 1.03 | 0.97 | 1.09 | 0.381 |
| Area level Index of Relative Socio-economic Disadvantage (IRSD) | | |  |  |  |  |  |  |
| Least disadvantage Quintiles 1 to 4 | 1.00 | Reference | |  | 1.00 | Reference | |  |
| Most disadvantage Quintile 5 | 1.36 | 0.74 | 2.51 | 0.101 | 0.92 | 0.86 | 0.97 | 0.01 |
| Comorbid conditions (Elixhauser) |  |  |  |  |  |  |  |  |
| 0 to 2 conditions | 1.00 | Reference | |  | 1.00 | Reference | |  |
| 3 or more conditions | 0.97 | 0.48 | 1.96 | 0.935 | 0.96 | 0.87 | 1.06 | 0.442 |
| GP consults |  |  |  |  |  |  |  |  |
| 0 to 13 consults | 1.00 | Reference | |  | 1.00 | Reference | |  |
| 14 or more consults | 1.03 | 0.55 | 1.93 | 0.926 | 1.07 | 0.99 | 1.15 | 0.099 |

|  | **h. Prostate** | |  |  |  |  |  |  |
| --- | --- | --- | --- | --- | --- | --- | --- | --- |
|  | **Aboriginal cohort** | | | | **non-Aboriginal cohort** | | | |
|  | Odds Ratio (adjusted) | Lower 95% Confidence Interval | Upper 95% Confidence Interval | p | Odds Ratio (adjusted) | Lower 95% Confidence Interval | Upper 95% Confidence Interval | p |
| **Main variable 1: 3 or more comorbid conditions** | |  |  |  |  |  |  |  |
| Age group at diagnosis |  |  |  |  |  |  |  |  |
| 50 to 69 years | 1.00 | Reference | |  | 1.00 | Reference | |  |
| 70 or more years | 2.19 | 1.33 | 3.61 | 0.002 | 5.06 | 4.67 | 5.49 | 0.000 |
| Area level Index of Relative Socio-economic Disadvantage (IRSD) | | |  |  |  |  |  |  |
| Least disadvantage Quintiles 1 to 4 | 1.00 | Reference | |  | 1.00 | Reference | |  |
| Most disadvantage Quintile 5 | 2.09 | 1.16 | 3.78 | 0.015 | 1.28 | 1.19 | 1.37 | 0.000 |
| **Main variable 2: 14 or more GP consults** |  |  |  |  |  |  |  |  |
| Age group at diagnosis |  |  |  |  |  |  |  |  |
| 50 to 69 years | 1.00 | Reference | |  | 1.00 | Reference | |  |
| 70 or more years | 1.85 | 1.37 | 2.48 | 0.000 | 2.66 | 2.57 | 2.76 | 0.000 |
| Sex |  |  |  |  |  |  |  |  |
| Male |  |  | |  |  |  | |  |
| Female |  |  |  |  |  |  |  |  |
| Area level Index of Relative Socio-economic Disadvantage (IRSD) | | |  |  |  |  |  |  |
| Least disadvantage Quintiles 1 to 4 | 1.00 | Reference | |  | 1.00 | Reference | |  |
| Most disadvantage Quintile 5 | 0.97 | 0.72 | 1.32 | 0.867 | 1.10 | 1.07 | 1.14 | 0.000 |
| Comorbid conditions (Elixhauser) |  |  |  |  |  |  |  |  |
| 0 to 2 conditions | 1.00 | Reference | |  | 1.00 | Reference | |  |
| 3 or more conditions | 6.77 | 3.93 | 11.68 | 0.000 | 3.60 | 3.36 | 3.86 | 0.000 |
| **Key outcome: Local-stage at diagnosis** |  |  |  |  |  |  |  |  |
| Age group at diagnosis |  |  |  |  |  |  |  |  |
| 50 to 69 years | 1.00 | Reference | |  | 1.00 | Reference | |  |
| 70 or more years | 0.79 | 0.61 | 1.03 | 0.088 | 0.61 | 0.59 | 0.63 | 0.000 |
| Sex |  |  |  |  |  |  |  |  |
| Male |  |  | |  |  |  | |  |
| Female |  |  |  |  |  |  |  |  |
| Area level Index of Relative Socio-economic Disadvantage (IRSD) | | |  |  |  |  |  |  |
| Least disadvantage Quintiles 1 to 4 | 1.00 | Reference | |  | 1.00 | Reference | |  |
| Most disadvantage Quintile 5 | 0.86 | 0.66 | 1.11 | 0.002 | 0.81 | 0.79 | 0.84 | 0.00 |
| Comorbid conditions (Elixhauser) |  |  |  |  |  |  |  |  |
| 0 to 2 conditions | 1.00 | Reference | |  | 1.00 | Reference | |  |
| 3 or more conditions | 0.70 | 0.42 | 1.18 | 0.180 | 0.69 | 0.65 | 0.74 | 0.000 |
| GP consults |  |  |  |  |  |  |  |  |
| 0 to 13 consults | 1.00 | Reference | |  | 1.00 | Reference | |  |
| 14 or more consults | 1.34 | 0.99 | 1.79 | 0.055 | 1.00 | 0.96 | 1.04 | 0.977 |

|  | **i. Head & neck** |  |  |  |  |  |  |  |
| --- | --- | --- | --- | --- | --- | --- | --- | --- |
|  | **Aboriginal cohort** | | | | **non-Aboriginal cohort** | | | |
|  | Odds Ratio (adjusted) | Lower 95% Confidence Interval | Upper 95% Confidence Interval | p | Odds Ratio (adjusted) | Lower 95% Confidence Interval | Upper 95% Confidence Interval | p |
| **Main variable 1: 3 or more comorbid conditions** | |  |  |  |  |  |  |  |
| Age group at diagnosis |  |  |  |  |  |  |  |  |
| 50 to 69 years | 1.00 | Reference | |  | 1.00 | Reference | |  |
| 70 or more years | 1.60 | 0.73 | 3.51 | 0.243 | 2.09 | 1.83 | 2.37 | 0.000 |
| Area level Index of Relative Socio-economic Disadvantage (IRSD) | | |  |  |  |  |  |  |
| Least disadvantage Quintiles 1 to 4 | 1.00 | Reference | |  | 1.00 | Reference | |  |
| Most disadvantage Quintile 5 | 1.83 | 0.67 | 4.98 | 0.235 | 1.28 | 1.12 | 1.45 | 0.000 |
| **Main variable 2: 14 or more GP consults** |  |  |  |  |  |  |  |  |
| Age group at diagnosis |  |  |  |  |  |  |  |  |
| 50 to 69 years | 1.00 | Reference | |  | 1.00 | Reference | |  |
| 70 or more years | 3.18 | 1.67 | 6.06 | 0.000 | 2.25 | 2.05 | 2.48 | 0.000 |
| Sex |  |  |  |  |  |  |  |  |
| Male | 1.00 | Reference | |  | 1.00 | Reference | |  |
| Female | 1.07 | 0.53 | 2.15 | 0.855 | 1.21 | 1.09 | 1.34 | 0.000 |
| Area level Index of Relative Socio-economic Disadvantage (IRSD) | | |  |  |  |  |  |  |
| Least disadvantage Quintiles 1 to 4 | 1.00 | Reference | |  | 1.00 | Reference | |  |
| Most disadvantage Quintile 5 | 1.48 | 0.68 | 3.24 | 0.326 | 1.01 | 0.92 | 1.11 | 0.879 |
| Comorbid conditions (Elixhauser) |  |  |  |  |  |  |  |  |
| 0 to 2 conditions | 1.00 | Reference | |  | 1.00 | Reference | |  |
| 3 or more conditions | 2.58 | 1.17 | 5.68 | 0.019 | 2.65 | 2.32 | 3.04 | 0.000 |
| **Key outcome: Local-stage at diagnosis** |  |  |  |  |  |  |  |  |
| Age group at diagnosis |  |  |  |  |  |  |  |  |
| 50 to 69 years | 1.00 | Reference | |  | 1.00 | Reference | |  |
| 70 or more years | 1.89 | 1.00 | 3.57 | 0.051 | 1.14 | 1.04 | 1.24 | 0.004 |
| Sex |  |  |  |  |  |  |  |  |
| Male | 1.00 | Reference | |  | 1.00 | Reference | |  |
| Female | 1.01 | 0.53 | 1.94 | 0.97 | 1.15 | 1.05 | 1.26 | 0.00 |
| Area level Index of Relative Socio-economic Disadvantage (IRSD) | | |  |  |  |  |  |  |
| Least disadvantage Quintiles 1 to 4 | 1.00 | Reference | |  | 1.00 | Reference | |  |
| Most disadvantage Quintile 5 | 0.48 | 0.25 | 0.90 | 0.243 | 0.85 | 0.79 | 0.93 | 0.00 |
| Comorbid conditions (Elixhauser) |  |  |  |  |  |  |  |  |
| 0 to 2 conditions | 1.00 | Reference | |  | 1.00 | Reference | |  |
| 3 or more conditions | 0.64 | 0.26 | 1.56 | 0.329 | 0.67 | 0.58 | 0.77 | 0.000 |
| GP consults |  |  |  |  |  |  |  |  |
| 0 to 13 consults | 1.00 | Reference | |  | 1.00 | Reference | |  |
| 14 or more consults | 1.96 | 1.01 | 3.80 | 0.047 | 1.14 | 1.03 | 1.26 | 0.009 |

**Supplementary Table S3: Multivariable model of causal influences on diagnosis of local cancer among Aboriginal and non-Aboriginal cohorts using continuous measures for age, comorbidities, GP consults and all quintiles of disadvantage**

|  |  | **Aboriginal cohort** | | | | **non-Aboriginal cohort** | | | |
| --- | --- | --- | --- | --- | --- | --- | --- | --- | --- |
| **DV is local stage (0=not local stage; 1= Local stage)** | | | | |  |  |  |  |  |
|  |  | Odds Ratio (adjusted) | Lower 95% CI | Upper 95% CI | p | Odds Ratio (adjusted) | Lower 95% CI | Upper 95% CI | p |
| Age |  |  |  |  |  |  |  |  |  |
|  |  | 0.99 | 0.98 | 1.00 | 0.047 | 0.98 | 0.98 | 0.98 | 0.000 |
| Sex |  |  |  |  |  |  |  |  |  |
|  | Male | 1.00 | Reference | | | 1.00 | Reference | | |
|  | Female | 0.80 | 0.70 | 0.91 | 0.001 | 0.97 | 0.95 | 0.98 | 0.000 |
| Area level socio-economic disadvantage (IRSD) | | | | |  |  |  |  |  |
|  | Q1 - Least | 1.37 | 1.04 | 1.81 | 0.025 | 1.42 | 1.40 | 1.45 | 0.000 |
|  | Q2 | 1.37 | 1.09 | 1.72 | 0.007 | 1.25 | 1.22 | 1.28 | 0.000 |
|  | Q3 | 1.15 | 0.95 | 1.40 | 0.149 | 1.15 | 1.13 | 1.83 | 0.000 |
|  | Q4 | 1.10 | 0.93 | 1.30 | 0.130 | 1.07 | 1.04 | 1.10 | 0.000 |
|  | Q5 - Most | 1.00 | Reference | | | 1.00 | Reference | | |
| Comorbid condition numbers (Elixhauser) | | | |  |  |  |  |  |  |
|  |  | 0.89 | 0.85 | 0.92 | 0.000 | 0.88 | 0.88 | 0.89 | 0.000 |
| GP consults | |  |  |  |  |  |  |  |  |
|  |  | 1.01 | 1.00 | 1.02 | 0.001 | 1.00 | 1.00 | 1.00 | 0.048 |
